# RNF8 has both KU-dependent and independent roles in chromosomal break repair

**DOI:** 10.1093/nar/gkaa380

**Published:** 2020-05-19

**Authors:** Linda Jillianne Tsai, Felicia Wednesday Lopezcolorado, Ragini Bhargava, Carlos Mendez-Dorantes, Eva Jahanshir, Jeremy M Stark

**Affiliations:** 1 Department of Cancer Genetics and Epigenetics, Beckman Research Institute of the City of Hope, Duarte, CA 91010, USA; 2 Irell and Manella Graduate School of Biological Sciences, Beckman Research Institute of the City of Hope, Duarte, CA 91010, USA

## Abstract

Chromosomal double strand breaks (DSBs) can initiate several signaling events, such as ubiquitination, however the precise influence of such signaling on DSB repair outcomes remains poorly understood. With an RNA interference screen, we found that the E3 ubiquitin ligase RNF8 suppresses a deletion rearrangement mediated by canonical non-homologous end joining (C-NHEJ). We also found that RNF8 suppresses EJ without insertion/deletion mutations, which is a hallmark of C-NHEJ. Conversely, RNF8 promotes alternative EJ (ALT-EJ) events involving microhomology that is embedded from the edge of the DSB. These ALT-EJ events likely require limited end resection, whereas RNF8 is not required for single-strand annealing repair involving extensive end resection. Thus, RNF8 appears to specifically facilitate repair events requiring limited end resection, which we find is dependent on the DSB end protection factor KU. However, we also find that RNF8 is important for homology-directed repair (HDR) independently of KU, which appears linked to promoting PALB2 function. Finally, the influence of RNF8 on EJ is distinct from 53BP1 and the ALT-EJ factor, POLQ. We suggest that RNF8 mediates both ALT-EJ and HDR, but via distinct mechanisms, since only the former is dependent on KU.

## INTRODUCTION

Chromosomal double strand breaks (DSBs) can be repaired by a variety of pathways. For example, end joining (EJ) repair can be mediated by canonical non-homologous EJ (C-NHEJ) or alternative EJ (ALT-EJ), which result in distinct repair outcomes (1–4). ALT-EJ causes deletion mutations with microhomology at the repair junction (5–9). Conversely, C-NHEJ can specifically mediate repair without requiring use of microhomology (4,10). DSBs can also be repaired by homologous recombination, such as homology-directed repair (HDR), which involves RAD51-dependent invasion of a homologous template for gene conversion (11). HDR that uses the identical sister chromatid as the template can restore the original sequence, whereas HDR that uses a non-identical sequence, such an exogenous template DNA, can alter the sequence (11,12). Defining the factors that mediate these specific DSB repair outcomes provides insight into mechanisms of gene editing, as well as the cellular response to clastogens.

Factors that affect repair outcomes include those that mediate DNA damage response (DDR) signaling. In particular, the ATM kinase catalyzes numerous phosphorylation events in response to DSBs, which appear important both to regulate repair, and initiate cell-cycle checkpoint responses (13). A key substrate of ATM is the histone variant H2AX (14,15), which when phosphorylated at serine 139 (i.e. γH2AX), creates a binding site for MDC1 (16). MDC1 interacts with several DDR factors including NBS1 (17–19) and the E3 ubiquitin ligase RNF8 (20–22). Since NBS1 is important for activation of the ATM kinase, the recruitment of NBS1 via MDC1 likely causes amplification of this signaling cascade (17–19).

The recruitment of RNF8 to DSBs initiates ubiquitin signaling that is critical to the activation and maintenance of the DDR. RNF8 contains two conserved domains: an FHA (Forkhead-associated) domain at the N-terminus, which interacts with phosphorylated MDC1 and is required for RNF8 recruitment to DSBs, and a RING (Really Interesting New Gene) finger E3 ubiquitin ligase domain at the C-terminus (20–24). Ubiquitin conjugation involves an activating enzyme (E1), a conjugating enzyme (E2) and a ligase (E3) that ultimately transfers the activated ubiquitin to a substrate lysine (K) residue, via an isopeptide bond with the C-terminus of ubiquitin (23,25). The lysine residues on ubiquitin itself can also serve as substrates for ubiquitination, causing polyubiquitin chains that are denoted by the lysine residue on ubiquitin that is the substrate (e.g. K11, K48 or K63 polyubiquitination) (23,25). RNF8 has been shown to promote several types of polyubiquitination, which appear to be specified by the E2 partner enzyme, i.e. with UBC13, UBCH8 and UBE2S to form K63, K48, and K11 polyubiquitin, respectively (20–24,26–30). These polyubiquitin chains have distinct functions, in that K48 polyubiquitination often targets the substrate protein for proteolysis, whereas K63 and K11 appear to initiate signaling events (20–30). The substrates of RNF8 include the protein L3MBT2L (31), and histone proteins (e.g. K11 polyubiquitination on H2A/H2AX) (26,32). Regarding the former, RNF8-mediated K63 polyubiquitination of L3MBT2L appears important for recruitment to DSBs of another RING finger E3 ligase, RNF168 (31), which amplifies the K63 polyubiquitin signal at DSBs and catalyzes ubiquitination of H2A/H2AX at K13/K15 (33–37).

Such ubiquitination that is dependent on RNF8 and RNF168 appears to mediate recruitment of several other DDR factors to DSBs, including BRCA1, 53BP1 and PALB2 (33–36). BRCA1 is important for HDR via promoting CtIP-dependent DSB end resection to form 3′ ssDNA that is the substrate for the RAD51 recombinase (38–40). BRCA1 is also important to promote recruitment of PALB2 to DNA damage, which is critical for HDR via its role in recruiting the RAD51-mediator, BRCA2 (36,41–43). 53BP1 is recruited to multimeric domains at DSBs, and is important to suppress HDR in BRCA1-deficient cells, as well as specific EJ events (i.e. class switch recombination, and fusion of deprotected telomeres) (44–49). RNF8 can also target the C-NHEJ factor KU (KU70/KU80) for ubiquitination, although whether RNF8 affects the dynamics of KU association with DSBs is controversial (29,50–51).

While such DDR signaling events have been well-established, their influence on discrete DSB repair outcomes remains to be defined. In particular, while RNF8 has been shown to promote HDR (35,52–53), its influence on EJ has been unclear. Through a targeted RNA interference (RNAi) screen, we identified RNF8, along with NBS1 and BRCA1, as important to suppress a deletion rearrangement mediated by C-NHEJ. Beginning with this observation, we sought to define the influence of RNF8 on distinct EJ outcomes. We found that RNF8 suppresses EJ of Cas9-induced DSBs without insertion or deletion (indel) mutations, which is a hallmark of C-NHEJ (54–56). Conversely, RNF8 promotes ALT-EJ involving microhomology embedded from the edge of the DSB, which likely requires limited end resection to reveal the microhomology. In contrast, RNF8 appears dispensable for a sub-type of homologous recombination (single-strand annealing, SSA) that requires extensive end resection. Importantly, the influence of RNF8 on ALT-EJ is dependent upon KU70. In contrast, RNF8 mediates HDR independently of KU, and its role in this repair event appears linked to promoting PALB2 function. We also find that the role of RNF8 on EJ is distinct from 53BP1 and the ALT-EJ factor POLQ. Thus, we suggest that RNF8 mediates both HDR and ALT-EJ, but via distinct mechanisms.

## MATERIALS AND METHODS

### Cell lines and plasmids

The reporter assay plasmids DR-GFP, EJ6-GFP, EJ2-GFP, EJ7-GFP, 4-μHOM and RMD-GFP, each of which are within a targeting vector for the *Pim1* gene, were previously described (8,54,57–59). All sgRNA/Cas9 plasmids are derived from px300 (Addgene 42230) (60), and the sgRNA sequences for targeting DSBs in the reporter assays were previously described (54,57,61). The pCAGGS-I-SceI (pCBASce), pCAGGS-KU70, pCAGGS-TREX2, pCAGGS-NZE-GFP (GFP transfection efficiency control), pgk-puro and POLQ expression vectors were previously described (8,62–63). The 3xFlag-RNF8 (3xF-RNF8) mouse expression vector was generated by inserting a gBLOCK fragment (Integrated DNA Technologies) into pCAGGS-BSKX, which is the control empty expression vector (EV) (63). The C406S mutant version of this expression vector was generated using the Quikchange kit (Agilent). The RNF8 human expression vector was previously described (61), as was the HEK293 EJ2-GFP cell line (8), and the control EV, pCMV6-XL5 (64). The pCAGGS-53BP1 (human) expression vector was generated from N-Myc-53BP1 WT pLPC-Puro (Addgene 19836) (47). The V5-FHA-PALB2 and V5-PALB2 expression vectors were generated from the pCAGGS-BSKX vector with the V5 tag sequence inserted, a plasmid with human PALB2 (Addgene 71114) (65) and polymerase chain reaction (PCR) amplification of the RNF8 FHA domain (human) from the PALB2-FHA plasmid (36), using these primers: 5′ CAGAAGATCTATGGGGGAGCCCGGCTTCTTCGTCACAG, and 5′ ACAGCCCGGGAGGCTCGTCCATTTCATCCAAACTGAATTTCCTT.

The WT, *Ku70-/-* and *Polq-/-* mouse embryonic stem cell (mESC) lines with each of the DSB reporter assays integrated into the *Pim1* locus, and the *53bp1-/-* mESC line, were previously described (6,54,57). To generate the *Rnf8-/-* mESC line from WT, these two sgRNA sequences were introduced into px330: 5′ GACACTAAGTCCCCGTCCTA and 5′ GCAGGGTTATTGCATCCGTAA. Cells on a 24-well dish were transfected with these two RNF8 sgRNA plasmids (200 ng each), along with 60 ng of dsRED plasmid and were subsequently sorted for dsRED+ cells as described previously (6), plated at low density and screened for a deletion in *Rnf8*, using these primers: mRNF8primer1 5′ GTGTTTGCAGCTGGTTGGTA, mRNF8primer2 5′ GAGCCAGACTCTCCCAGTCTT and mRNF8primer3 5′ CTCTGTGGAGGCATGAAGGT. RT-PCR analysis of RNF8 was performed using RNA isolated with the RNeasy Plus Minikit (Qiagen 74134) and treated with M-MLV Reverse Transcriptase (Promega M170A) to generate cDNA, prior to PCR amplification with primers for RNF8 (5′ ATTAAGTTGCGCGAGAGGAA, and 5′ AGCTCGTTCTCCAGCAAGTC), and for Actin (5′ GGCTGTATTCCCCTCCATCG, 5′ TCTCCAGGGAGGAAGAGGAT). The generation of the *53bp1*-/-*Rnf8*-/- mESC line, and attempts to generate a homozygous deletion of *Rnf8* in the *Polq-/-* mESC line, used the same sgRNA plasmids and PCR screening as for the *Rnf8*-/- mESCs.

To generate the *RNF8*-*KO* HEK293 EJ2-GFP cell lines, these two sgRNA sequences were inserted into px330: 5′ GAGCGCGTCTGGAACCTTTA, and 5′ gCATCCAACTTGGAGTGCCTC. The HEK293 EJ2-GFP cell lines were seeded on a 6-well plate, transfected with 400 ng of each RNF8 sgRNA plasmid along with 150 ng of dsRED plasmid, and were subsequently sorted for dsRED+ cells, plated at a low density and screened for a deletion in *RNF8* using these primers RNF8hPCRdn2 5′ GGAGCCCCTGTGGTCTTACT and RNF8hPCRup2 5′ GCAGCAGGAGAGAGATTCCTT.

The *Ku70* gene was disrupted in the *Polq-/-* and *Rnf8-/-* mESC line using two sgRNAs targeting *Ku70* that were cloned into px330: 5′ GCCATGGGGGTCGTCTTCAT, and 5′ GACCCATGGCCAACCGTGTCT. These two sgRNA/Cas9 plasmids (200 ng each) targeting *Ku70* were co-transfected with the dsRED plasmid (60 ng) into the *Rnf8-/-* cell line on a 24-well dish, cells were sorted for dsRED+ and plated at low density to isolate colonies. The procedure was similar for the *Polq-/-* line, except scaled to a 12-well dish and using 400 ng of each sgRNA/Cas9 plasmid and 100 ng dsRED plasmid. Reporter assay cassettes were integrated into the *Pim1* locus of each cell line using electroporation of linearized reporter plasmids, selection of targeted clones with hygromycin treatment and screening for *Pim1* targeting using PCR analysis, as described (54).

### DSB reporter assays

The day before each plasmid transfection, mESCs, or HEK293 cells, were seeded at a cell density of 0.5 × 10^5^ cells per well of a 24-well dish. The plasmid amounts used were 200 ng of each sgRNA/Cas9 plasmid, 200 ng of I-SceI (pCBASce) plasmid, 200 ng of the pCAGGS-TREX2 plasmids and 100 ng of complementation vectors. In addition, for each well, control EV was added to bring the total plasmid amount to 700 ng for mESCs and 600 ng for HEK293 cells. Plasmids were transfected with Lipofectamine 2000 (Thermo Fisher, 2.1 μl for mESCs, 2 μl for HEK293) in 0.5 ml of antibiotic-free media. For siRNA experiments, cells were seeded on a mixture of 3.75 pmol of siRNA with 1 μl of RNAiMAX (Thermo Fisher). For the plasmid transfection on the following day, 3.75 pmol of siRNA was included, along with the same plasmid amounts as described above, however, using a total of 600 ng of plasmid, and 2 μl of Lipofectamine 2000. The siRNAs were acquired from Dharmacon, and the catalog numbers are shown in Supplementary Table S1, except for the nontargeting siCTRL (Dharmacon, D-001810-01) and siCtIP (Dharmacon, equal amounts of four siRNAs, D-055713-14, D-055713-15, D-055713-16 and D-055713-17). For the immunoblotting and immunofluorescence analyses, the plating and transfection reactions were scaled up 4-fold for mESCs, and 2-fold for HEK293 cells. To examine Cas9 expression by immunoblotting, the transfections were scaled 2-fold but included 200 ng of pgk-puro plasmid, cells were treated with puromycin the day after transfection to enrich for transfected cells and cultured for two more days, such that the total timing matched the reporter assays. Namely for reporter assays, cells were analyzed 3 days post transfection by flow cytometry using a CyAn ADP Analyzer (Dako), as described (63). For the siRNA screen, GFP+ frequencies were not normalized to transfection efficiency, but rather were normalized to parallel siCTRL treatments. All other experiments with siRNA were normalized to both transfection efficiency and siCTRL. Finally, experiments without siRNA were normalized to transfection efficiency. To normalize to transfection efficiency, a parallel transfection with a GFP expression vector was used (pCAGGS-NZE-GFP) (63).

### Immunoblotting and immunofluorescence analysis

For immunoblotting analysis, cells were lysed with NETN buffer (20  mM TRIS (pH 8.0), 100 mM NaCl, 1 mM ethylenediaminetetraacetic acid (EDTA), 0.5% IGEPAL, 1.25  mM dithiothreitol and Roche Protease Inhibitor), and followed by several freeze/thaw cycles. To generate cell extracts to probe for Cas9 (Flag), cells were lysed in ELB buffer (250 mM NaCI, 5 mM EDTA, 50 mM HEPES, 0.1% Ipegal, Roche protease inhibitor) with sonication (Qsonica, Q800R). Blots were probed with the following antibodies: CtIP (1:1000, Active Motif 61141); KU70 (1:1000, Cell Signaling D10A7); BRCA1 (1:500) (66); NBS1 (1:1000, Bethyl A300–284A); Flag-HRP (1:1000, Sigma Aldrich A8592); RNF8 (1:1000, Santa Cruz sc271462); 53BP1 (1:1000, Abcam Ab36823); V5 (1:250, Thermo Fisher R96125); Actin (1:3000, Sigma Aldrich A2066), and Tubulin (1:1000, Sigma Aldrich T9026). HRP-conjugated secondary antibodies (Abcam), and ECL reagent (Amersham Biosciences) was used to develop the immunoblotting signals.

For immunofluorescence, cells were seeded at a density of 2 × 10^5^ cells per 6-cm^2^ plate overnight. The next day, the plates were exposed to 6 Gy ionizing radiation (IR) (Gammacell 3000), cells were incubated for 4 h in media, were detached with trypsin and fixed in 2% paraformaldehyde in phosphate-buffered saline. Cells were affixed to slides using a Cytospin 4 (Thermo Fisher), permeabilized (0.5% Triton X-100) and treated with the following antibodies: 53BP1 (1:500, Abcam ab36823); γH2AX (1:500, Novus NB100-78356 for co-staining with 53BP1, and 1:250, Novus NBP1-19255 for co-staining with RAD51); RAD51 (1:250, Millipore PC130); AlexaFluor 488 (1:250, Invitrogen A11029); and AlexaFluor 568 (1:250, Invitrogen A11036), and stained with DAPI using Vectashield Mounting Medium (Vector Laboratories H1500). Images were acquired using the Zeiss Observer II with the 40× objective with the ZEN Black image acquisition software. Foci were counted using ImageJ.

### Cell-cycle analysis and clonogenic survival

For cell-cycle analysis, cells were treated with 10 μM bromodeoxyuridine (BrdU, Becton Dickinson, 51-2420KC) for 30 min, fixed with 70% Ethanol and stained with FITC-conjugated anti-BrdU antibody (BD Pharmingen 556028), propidium iodine (PI, Sigma Aldrich P4170) and RNase A (Sigma Aldrich 4642). BrdU and propidium iodide staining were evaluated using a CyAn ADP Analyzer (Dako). To examine cell-cycle phase post-transfection, cells were transfected as for the reporter assays, but scaled 2-fold and including 200 ng pgk-puro plasmid. These cells were treated the next day with puromycin (3 μg/ml) for 1 day to enrich for transfected cells prior to BrdU labeling and fixation. To examine clonogenic survival after IR treatment, cells were treated with either 0, 1.5 or 3 Gy of IR (Gammacell 3000), and seeded at low density to form colonies, which were fixed (10% acetic acid, 10% methanol), stained with 1% crystal violet and counted under the microscope with a 10× objective. To calculate the frequency of clonogenic survival, the number of colonies per well were normalized to the number of cells plated and this colony forming value for each treatment was divided by the mean value of the parallel untreated plates (0 Gy).

### Amplicon deep sequencing

To examine EJ junctions with the EJ6-GFP reporter, cells were seeded on a 6-well dish, transfected with 1 μg of each of the two sgRNA/CAS9 plasmids and EV with 10 μl of Lipofectamine 2000 (Thermo Fisher), cells were sorted to enrich for GFP+ and amplicons generated for deep sequencing using the HiSeq2500 platform (Illumina), and the reads were aligned to the reference sequence, as described previously (57). We also manually aligned the sequences ≥100 bp that were unaligned by this bioinformatics analysis, and that also had read frequencies of ≥0.01%. The sequencing reads of each amplicon (>170 000 reads per sample) were examined to determine the frequencies of distinct junction categories (i.e. no indel, deletion, insertion and complex, see ‘Results’ section). We note that some deletions with short insertions cannot be distinguished from deletions with point mutations by the bioinformatics analysis, and hence are grouped within the deletion category. Microhomology usage was examined manually for all sequences with read frequencies of ≥0.5% of total deletion reads. For each cell line, independent transfections were used to generate three independent amplicons, and the frequencies of junction categories from these replicates were used to calculate the mean and standard deviation.

### I-SceI site loss assay

Quantifying the loss of the I-SceI recognition site with the DR-GFP reporter assay was performed as described previously (63,67). Briefly, cells were transfected as for the DSB reporter assays, except scaled to a 12-well dish (i.e. 400 ng the sgRNA/Cas9 plasmid targeting the I-SceI site in DR-GFP, 1000 ng EV and 4.2 μl of Lipofectamine 2000). Control wells were left untransfected. After 3 days, genomic DNA was purified from the cells, which was PCR amplified with DRp1 5′CTGCTAACCATGTTCATGCC and DRp2 5′AAGTCGTGCTGCTTCATGTG, gel purified and digested with I-SceI (New England Biolabs), as previously described (63). The PCR used Platinum HiFi PCR Supermix (Thermofisher), and amplification conditions were 33 cycles of 94°C 45 s, 63°C 45 s, 68 °C 1 min. Band intensity was calculated with GelAnalyzer (ImageJ), and the frequencies of I-SceI site loss were determined as described (63).

## RESULTS

### RNAi screen for factors that inhibit a C-NHEJ-mediated deletion rearrangement

We sought to identify factors that affect distinct EJ outcomes mediated by C-NHEJ versus ALT-EJ. For this, we used a set of green fluorescent protein (GFP)-based reporter assays to examine such EJ outcomes, which we first validated using the C-NHEJ factor KU70, and the ALT-EJ factor CtIP. Each reporter assay uses the RNA guided nuclease Cas9 to induce one or more chromosomal DSBs, and is designed such that repair leading to a specific outcome generates a GFP+ expression cassette. All reporters are integrated into the *Pim1* locus of chromosome 17 in mESCs.

**Figure 1.**
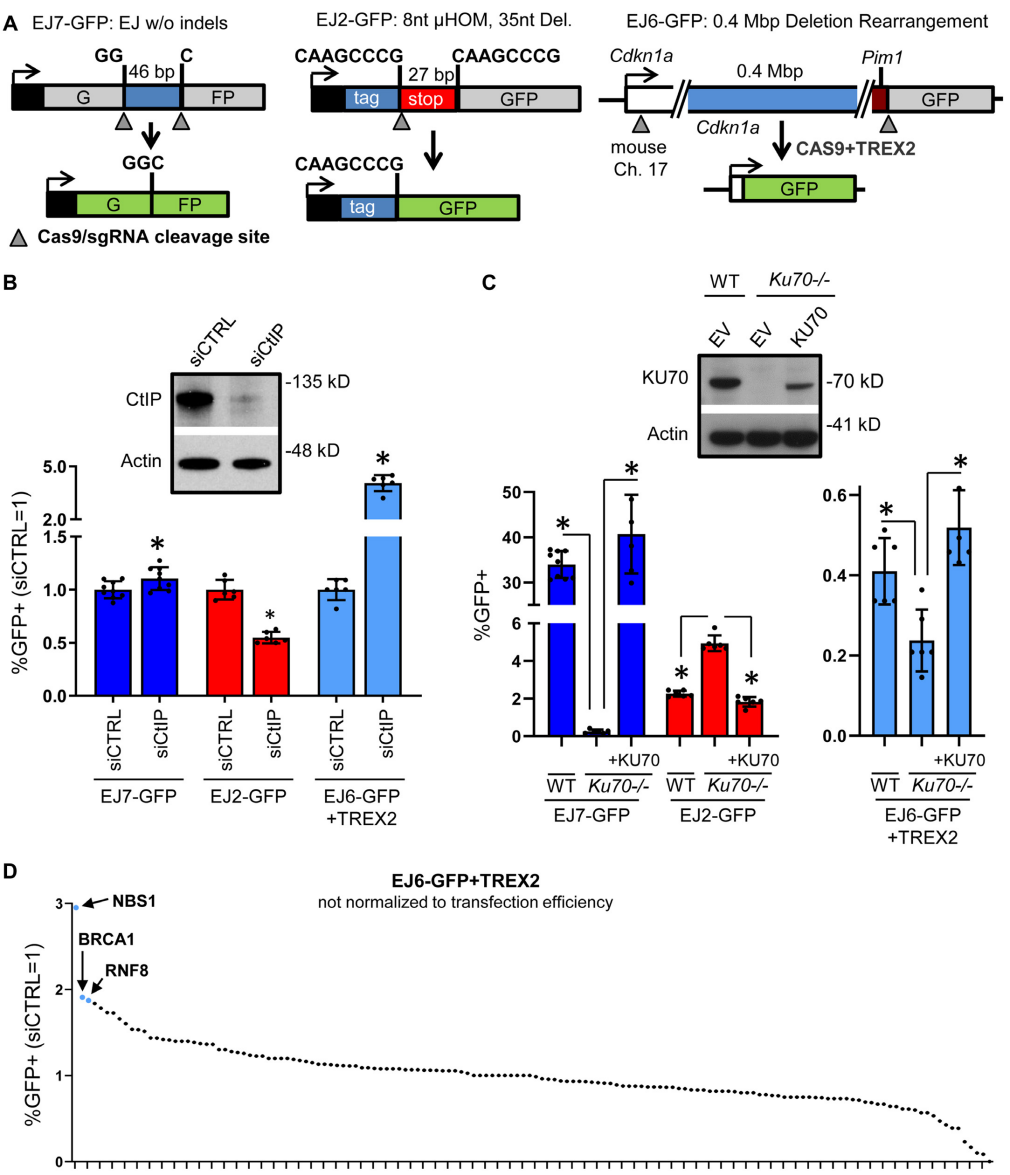
RNAi screen for factors that inhibit a C-NHEJ-mediated deletion rearrangement. (**A**) Shown are diagrams of three EJ reporter assays that use the RNA-guided nuclease Cas9 to induce DSBs. With each assay, a specific repair event restores GFP+ expression. EJ7-GFP measures EJ between two tandem DSBs that causes loss of the fragment between the DSBs, but without further indel mutations. EJ2-GFP measures a deletion EJ event that uses flanking 8-nt of microhomology to restore GFP. EJ6-GFP measures a large deletion rearrangement between two DSBs separated by 0.4 Mbp. Shown is a version of the EJ6-GFP assay that uses overexpression of the 3′ exonuclease TREX2. Each GFP reporter is integrated into chromosome 17 of mESCs at the *Pim1* locus. (**B**) Influence of CtIP on distinct EJ events. Shown are the GFP+ frequencies for the assays in (A) in WT mESCs treated with a pool of four siRNAs targeting CtIP (siCtIP) or non-targeting siRNA (siCTRL). Error bars represent SD. *N* = 6 for EJ6-GFP+TREX2, and EJ2-GFP. *N* = 9 for EJ7-GFP. (*) *P* < 0.001, using an unpaired *t*-test with the Holm–Sidak correction. Also shown is an immunoblot analysis for CtIP in WT mESCs treated with either siCTRL or siCtIP. Actin signals shown for loading control. (**C**) Influence of KU70 on distinct EJ events. Shown are the GFP+ frequencies for the EJ reporter assays shown in (A) in WT mESCs, *Ku70-/-* mESCs and *Ku70-/-* mESCs transfected with a KU70 expression vector. Error bars represent SD. *N* = 6, except *N* = 9 for WT EJ7-GFP. (*) *P* ≤ 0.006 using unpaired multiple *t*-tests with the Holm–Sidak correction. Also shown are immunoblot signals for KU70 and Actin for WT mESCs, *Ku70-/-* mESCs and *Ku70-/-* mESCs transfected with an expression vector for KU70, or only control empty vector (EV). (**D**) Effects of siRNAs targeting 148 factors on the EJ6-GFP/TREX2 assay. Each of the points represents the effect of an RNAi reagent targeting a single gene (pool of four siRNAs per gene) on the EJ6-GFP+TREX2 assay. Repair frequencies were normalized to siCTRL (siCTRL = 1), but these screening experiments were not normalized to transfection efficiency. The siRNAs causing the three highest frequencies are highlighted. *N* ≥ 2.

To examine C-NHEJ, we used EJ7-GFP, which is designed to measure EJ between two DSBs without insertion and/or deletion (indel) mutations (Figure 1A) (54). In this reporter, the GFP cassette is interrupted by a 46-bp insertion. Single guide RNAs (sgRNAs) and Cas9 are used to target two DSBs to precisely excise this insertion. Cas9 largely generates blunt ended DSBs (68), such that EJ that uses the distal DSB ends without processing prior to ligation (i.e. without causing indel mutations) restores GFP+. Our laboratory previously found that loss of the end resection factor CtIP causes a modest increase in EJ without indels, whereas several C-NHEJ factors, including KU70, are absolutely required for this event (54). We have confirmed these findings using siRNA depletion of CtIP (siCtIP) in mESCs, which caused a significant, but modest increase in such EJ compared to non-targeting siRNA (siCTRL, Figure 1B). We also confirmed that loss of KU70 abolishes this EJ event, based on a comparison of *Ku70-/-* mESCs versus these cells with transient expression of KU70, as well as WT mESCs (Figure 1C). In summary, C-NHEJ is required for EJ between two Cas9-induced DSBs without indel mutations, which is measured by the EJ7-GFP reporter.

To examine ALT-EJ, we used the EJ2-GFP reporter that has an N-terminal tag sequence separated from the GFP sequence by an insert containing stop codons in all three reading frames (Figure 1A) (8). This insert sequence is flanked by 8-nt of microhomology, such that a DSB repair event that uses this microhomology restores GFP+, and causes a 35-nt deletion. We used Cas9 with an sgRNA to target a DSB immediately downstream from the 5′ microhomology sequence, as previously described (57). We first examined the influence of CtIP using RNAi, and found that siCtIP caused a significant decrease in this repair event, compared to siCTRL (1.8-fold, Figure 1B). In contrast, loss of KU70 caused a substantial increase in this repair event (2.7-fold, *Ku70-/-* versus these cells with transient expression of KU70, and versus WT mESCs, Figure 1C). These findings are consistent with other reports that CtIP promotes ALT-EJ, whereas KU70 inhibits such repair (8,69).

Finally, we examined a deletion rearrangement event that is mediated by C-NHEJ, using a variant of the EJ6-GFP reporter. In this reporter, a promoter-less GFP cassette is located 0.4 megabase pairs (Mbp) downstream from the endogenous *Cdkn1a* promoter (57). We used sgRNAs/Cas9 to induce two DSBs: the first is downstream of the *Cdkn1a* promoter, and the second is upstream of the GFP coding sequence. EJ that uses the distal DSB ends causes a deletion rearrangement that places GFP downstream from the *Cdkn1a* promoter, thereby creating a GFP+ expression cassette. Our laboratory has found that this deletion rearrangement can be mediated by either C-NHEJ or ALT-EJ (57). However, including expression of the 3′ exonuclease TREX2 (70) causes an increased reliance on C-NHEJ for this rearrangement (57). We posit that TREX2-mediated degradation of 3′ ssDNA blocks the availability of such 3′ ssDNA for microhomology annealing to facilitate ALT-EJ. Thus, TREX2 expression likely disrupts ALT-EJ, thereby causing an increased reliance on C-NHEJ for the deletion rearrangement. Consistent with this notion, we confirmed here that loss of KU70 causes a significant reduction in the frequency of this deletion rearrangement (EJ6-GFP/TREX2, 2.2-fold, *Ku70-/-*versus these cells with transient expression of KU70, as well as WT mESCs, Figure 1C).

Additionally, our laboratory previously showed that disruption of the ATM kinase causes a marked increase in the frequency of deletion rearrangements with this assay (EJ6-GFP/TREX2), indicating that ATM suppresses C-NHEJ-mediated deletion rearrangements (57). To further examine the mechanism of these C-NHEJ-mediated deletion rearrangements, we examined the effect of CtIP and found that siCtIP caused a marked increase in these events (i.e. a 4.1-fold increase in the EJ6-GFP/TREX2 assay, Figure 1B). While the mechanism of these rearrangement events is likely complex, due to the combination of two Cas9 DSBs and TREX2 expression, the marked influence of CtIP on this assay indicated that it might be a useful screening tool to identify other factors that may have similar effects on EJ outcomes.

Accordingly, we performed an RNAi screen to identify factors with effects on EJ similar to CtIP, i.e. that cause an increase in C-NHEJ-mediated deletion rearrangements (EJ6-GFP/TREX2). For this, we developed a library of siRNAs targeting 148 genes. The 148 gene list was generated using a review article that summarized major pathways in the ATM DDR (113 of the genes) (13), which was supplemented with an additional 35 genes in the ATM DDR that were not listed in the review article and/or were identified from a literature search for NHEJ (Supplementary Table S1). Each siRNA reagent (a pool of four siRNAs per gene) was examined for effects on repair frequencies, relative to parallel experiments with non-targeting siCTRL. From this screen, we found siRNAs targeting three factors caused a marked increase in C-NHEJ-mediated deletion rearrangements: BRCA1, NBS1 and RNF8 (Figure 1D and Supplementary Table S1).

### BRCA1 and NBS1 promote ALT-EJ

**Figure 2.**
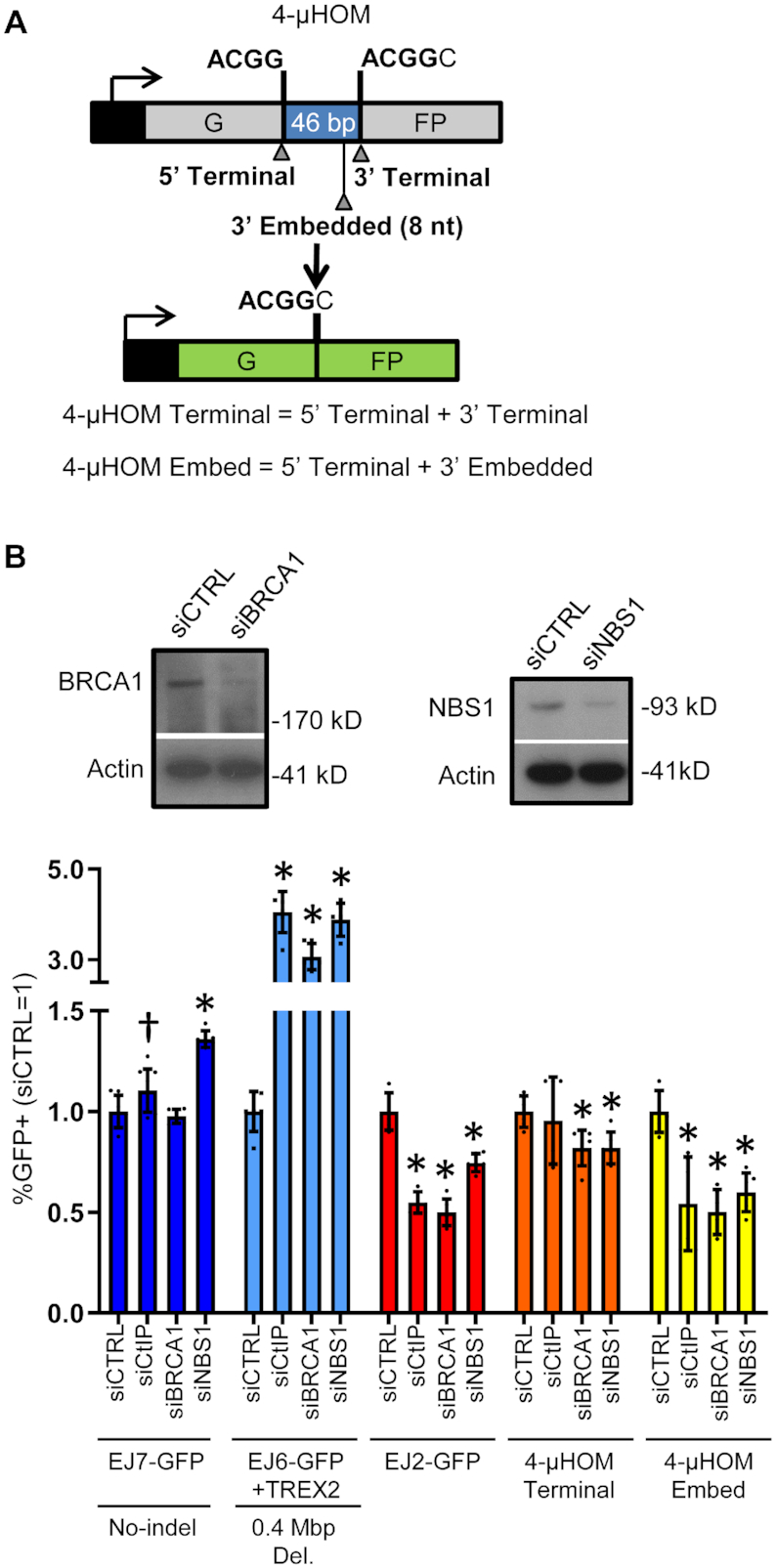
BRCA1 and NBS1 promote ALT-EJ. (**A**) Shown is a diagram for the 4-μHOM reporter for EJ, which is a variant of EJ7-GFP. This reporter contains two tandem repeats of four nucleotides (i.e. 4-nt of microhomology), which if used to bridge the ends during EJ, restores GFP+. Two DSBs are induced in the reporter: one DSB downstream from the 5′ microhomology, and a second DSB upstream from the 3′ microhomology. The second DSB can either be at the edge of the 3′ microhomology (4-μHOM Terminal), or 8-nt upstream (4-μHOM Embed). (**B**) CtIP, BRCA1 and NBS1 promote ALT-EJ. Shown are immunoblot signals of BRCA1 and NBS1, and Actin control, from cells treated with siRNAs targeting these factors, respectively, versus non-targeting siCTRL. Also shown are the GFP+ frequencies for several chromosomal reporter assays in WT mESCs (EJ7-GFP, EJ6-GFP+TREX2, EJ2-GFP, 4-μHOM Terminal and 4-μHOM Embed), treated with siRNAs targeting CtIP, BRCA1 and NBS1. GFP+ frequencies are normalized to transfection efficiency, and parallel treatments with siCTRL. Values for EJ7-GFP, EJ6-GFP+TREX2 and EJ2-GFP, for siCTRL and siCtIP are from Figure 1B and are shown here for comparison. Error bars represent SD. *N* = 6, except *N* = 9 for EJ7-GFP siCTRL and siCtIP. (†) *P* = 0.033 for siCTRL versus siCtIP using an unpaired *t*-test without correction. (*) *P* ≤ 0.008 versus siCTRL, using unpaired multiple *t*-tests with the Holm–Sidak correction.

We then sought to examine the influence of BRCA1, NBS1 and RNF8 on other EJ events. For this analysis, we included another published reporter assay, which is called 4-μHOM (Figure 2A) (54). This assay is a variant of the EJ7-GFP assay, but the insert sequence that disrupts GFP is flanked by 4-nt of microhomology. To examine EJ mediated by the 4-nt of microhomology, we induce two Cas9 DSBs. The 5′ DSB is targeted immediately downstream of the first sequence of microhomology. The 3′ DSB can be targeted either immediately upstream of the second sequence of microhomology (4-μHOM Terminal), or 8-nt upstream (4-μHOM Embed, to denote that the microhomology is embedded from the DSB end). Thus, the 4-μHOM Terminal event requires exposure of only the 4-nt at the edge of each DSB, while the 4-μHOM Embed event requires exposure of an additional 8-nt to uncover the second sequence of microhomology. These two EJ events appear to have distinct mechanistic requirements. Namely, our laboratory reported that CtIP is important to promote the 4-μHOM Embed event, but not the 4-μHOM Terminal event. We have confirmed these results here using siCtIP treatment (Figure 2B). In summary, the 4-μHOM assay can be used to examine EJ events mediated by 4-nt of microhomology that is either at the edge of the DSB, or embedded from the edge of the DSB by 8-nt, where only the latter of which is mediated by CtIP. Due to this differential requirement for the ALT-EJ factor CtIP, we suggest that the 4-μHOM Embed event is a measure of ALT-EJ, whereas the 4-μHOM Terminal event appears to be mediated by a distinct mechanism.

Using the above assays, we first examined the influence of RNAi depletion of NBS1 and BRCA1 on distinct EJ events. We validated that the siRNAs targeting NBS1 and BRCA1 cause depletion of the respective target protein, using immunoblot analysis (Figure 2B). For each siRNA experiment, we normalized GFP+ frequency to siCTRL, as well as transfection efficiency. For comparison, the GFP+ frequencies for siCTRL-treated cells for each of the reporter assays, along with transfection efficiency, without normalization, are provided in Supplementary Figure S2. Consistent with the screen results, depletion of NBS1 and BRCA1 caused a marked increase in C-NHEJ-mediated deletion rearrangements (EJ6-GFP/TREX2) (Figure 2B). NBS1 depletion also caused a significant increase in EJ without indels (1.4-fold, EJ7-GFP), whereas BRCA1 depletion had no obvious effect on such EJ (Figure 2B). In contrast, depletion of BRCA1 and NBS1 caused a significant reduction in ALT-EJ as measured by both the EJ2-GFP, and the 4-μHOM Embed assays (≥1.7-fold, Figure 2B). Finally, depletion of BRCA1 and NBS1 had only modest effects on the 4-μHOM Terminal assay (1.2-fold decrease, Figure 2B). Thus, BRCA1 and NBS1 appear particularly important for ALT-EJ, and also to suppress C-NHEJ-mediated deletion rearrangements. Furthermore, NBS1 suppresses EJ without indels, which is a hallmark of C-NHEJ.

### RNF8 promotes ALT-EJ

**Figure 3.**
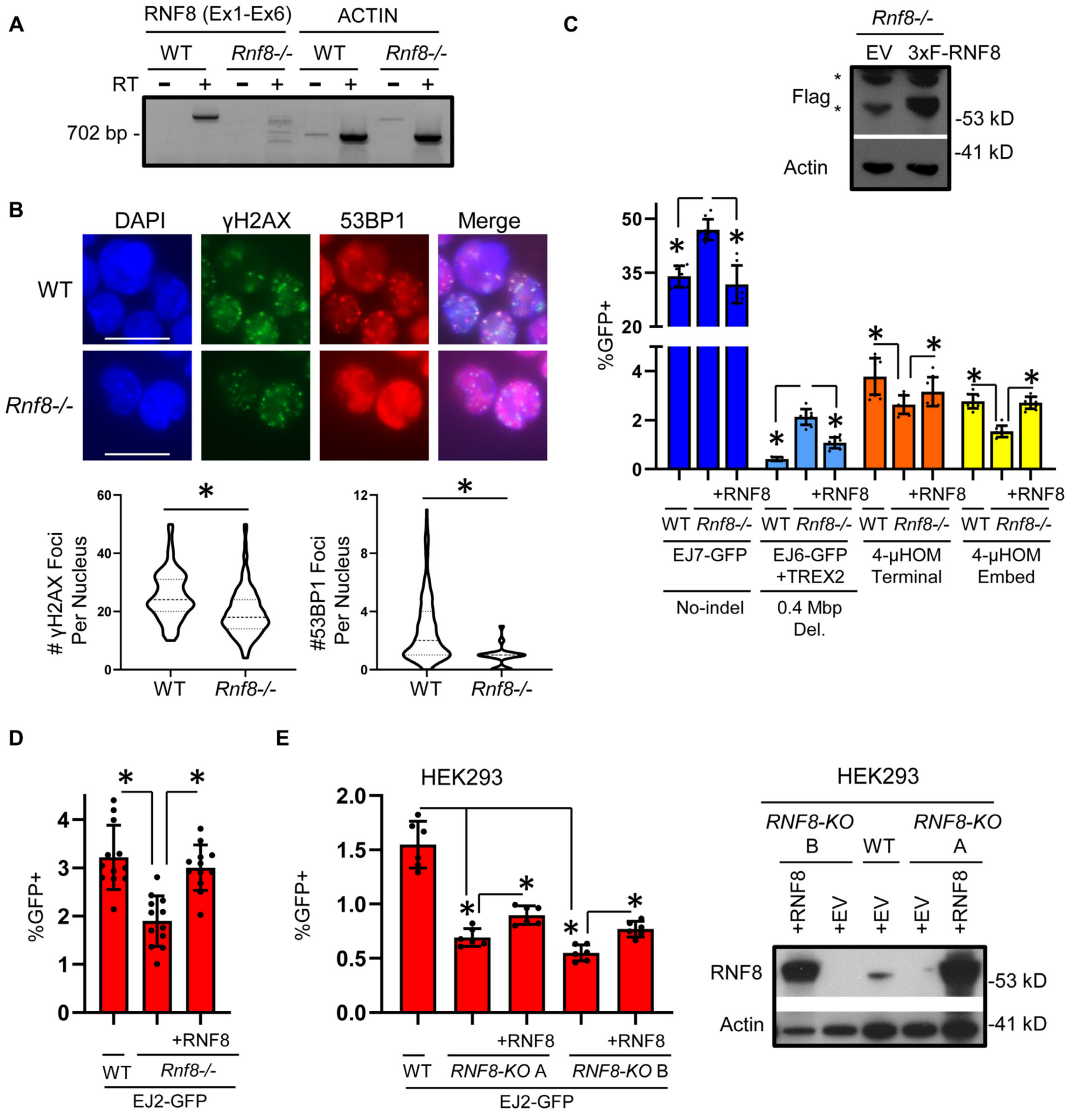
RNF8 promotes ALT-EJ. (**A**) RNA analysis of an *Rnf8-/-* mESC line generated using Cas9 and sgRNAs targeting *Rnf8*. Shown are RT-PCR amplification products for a region of the RNF8 mRNA from exons 1–6 (Ex1–Ex6), and Actin control, for RNA from WT and *Rnf8-/-* mESCs, treated with and without reverse transcriptase (RT). (**B**) The *Rnf8-/-* mESC line shows a defect in 53BP1 foci formation. Shown are representative fluorescent images of WT and *Rnf8-/-* mESCs treated with 6 Gy and recovered for 4 h, stained with DAPI and antibodies for γH2AX and 53BP1. Scale bar = 20 μm. Also shown are violin plots depicting the number of γH2AX foci per nucleus (left) and 53BP1 foci per nucleus (right) in WT and *Rnf8-/-* mESC. *N* = 120. (*) *P* < 0.001 for WT versus *Rnf8-/-* measuring γH2AX or 53BP1 foci using the Mann–Whitney test. (**C**) RNF8 promotes ALT-EJ, and inhibits EJ without indels (i.e. C-NHEJ). Shown are the GFP+ frequencies for several chromosomal reporter assays (EJ7-GFP, EJ6-GFP+TREX2, 4-μHOM Terminal and 4-μHOM Embed) in WT mESCs, *Rnf8-/-* mESCs and *Rnf8-/-* mESCs transfected with a 3×Flag-tagged RNF8 expression vector (3×F-RNF8). WT EJ7-GFP and EJ6-GFP+TREX2 values are from Figure 1C and shown here for comparison. As with all mESC experiments in this study, the reporters are integrated into chromosome 17 at the *Pim1* locus. Error bars represent SD. *N* ≥ 6. (*) *P* ≤ 0.038 using unpaired multiple *t*-tests with the Holm–Sidak correction. Also shown is an immunoblot staining for Flag, and Actin control, in *Rnf8-/-* mESCs transfected with and without 3×F-RNF8. (*) Non-specific bands. (**D**) RNF8 promotes ALT-EJ in mESCs, as measured by the EJ2-GFP reporter. Shown are GFP+ frequencies for EJ2-GFP in WT and *Rnf8-/-* mESCs with and without complementation vector. *N* = 12. Error bars represent SD. (*) *P* ≤ 0.001 for unpaired multiple *t*-tests with the Holm–Sidak correction. (**E**) RNF8 promotes ALT-EJ in HEK293 cells, as measured by the EJ2-GFP reporter. Two independent *RNF8* knockout (*RNF8-KO*) cell lines (*RNF8-KO* clones A and B) were generated in an HEK293 cell line with the EJ2-GFP reporter. Shown are GFP+ frequencies for the EJ2-GFP assay for these two cell lines with and without an RNF8 expression vector, as well as for the HEK293 WT cell line. *N* = 6. Error bars represent SD. (*) *P* ≤ 0.001766 for unpaired multiple *t*-tests with the Holm–Sidak correction. Also shown is an immunoblot analysis for RNF8, and Actin control, for the two *RNF8-KO* HEK293 cell lines transfected with RNF8, or only control EV, along with HEK293 WT cells transfected with EV.

The third factor identified in our screen was RNF8, which we studied by developing an *Rnf8-/-* mESC line. This cell line was generated by targeting two Cas9-induced DSBs in the *Rnf8* gene (one in exon 2, and the second in exon 3), which caused a deletion between the two DSBs, and resulted in a frame-shift (Supplementary Figure S1A). To validate loss of RNF8 expression in this cell line, we used RT-PCR to amplify a segment of the RNF8 mRNA from exons 1–6, and found that while this amplification product is readily detected in WT cells, it is absent in the *Rnf8-/-* mESC line (Figure 3A). Our attempts to detect mouse RNF8 protein with immunoblotting with commercial antibodies were unsuccessful. Thus, to further confirm loss of RNF8 function in the *Rnf8-/-* mESC line, we used a functional assay. Namely, RNF8 has been shown to be critical for recruitment of 53BP1 to DSBs (20–22). Thus, we examined formation of 53BP1 foci after IR treatment, and found that *Rnf8-/-* mESCs had a marked reduction in 53BP1 foci as compared to WT mESCs (Figure 3B). In contrast, γH2AX foci, which is a marker of DNA damage, were readily detectable in both cell lines, although *Rnf8-/-* mESCs showed a modest reduction versus WT (Figure 3B).

Using this *Rnf8-/-* mESC line, we examined the influence of RNF8 loss on EJ, by first integrating several DSB reporter assays into this line (EJ7-GFP, EJ6-GFP and 4-μHOM). For each assay, we compared the repair frequencies between *Rnf8-/-* versus WT, as well as *Rnf8-/-* mESCs co-transfected with a complementation vector for mouse RNF8 with a 3×Flag immunotag (3×F-RNF8). We found similar results with either comparison (i.e. *Rnf8-/-* versus WT, and *Rnf8-/-* versus complemented). Beginning with the C-NHEJ-mediated deletion rearrangement assay (EJ6-GFP/TREX2), we found that loss of RNF8 caused a marked increase in this event (5.2-fold, Figure 3C), which is consistent with the results of the siRNA screen (Figure 1D). Similarly, loss of RNF8 caused a significant increase in EJ without indels (1.4-fold, Figure 3C). In contrast, with the 4-μHOM assays, we found that loss of RNF8 caused a significant reduction in both 4-μHOM Embed, and 4-μHOM Terminal, although the fold-effect was diminished for the latter, (1.8- and 1.4-fold, respectively, Figure 3C). In summary, RNF8 appears to suppress C-NHEJ-mediated deletion rearrangements, and EJ without indels, which is a hallmark of C-NHEJ. In contrast, RNF8 appears to mediate ALT-EJ, as measured by the 4-μHOM Embed assay. Finally, RNF8 modestly promoted EJ involving terminal microhomology (4-μHOM Terminal assay).

We next tested whether RNF8 is also important for ALT-EJ in human cells, specifically HEK293 cells. For this, we used the EJ2-GFP reporter, described above (Figure 1A), since our laboratory had previously generated an HEK293 cell line with this reporter integrated (8). To enable a direct comparison between HEK293 and mESCs, we also tested the EJ2-GFP reporter in the *Rnf8-/-* mESCs, and found that similar to the 4-μHOM Embed assay, loss of RNF8 caused a significant reduction in ALT-EJ by the EJ2-GFP assay (1.7-fold), which was restored by transient expression of mouse RNF8 (3×F-RNF8, Figure 3D). Next, we used HEK293 EJ2-GFP cells to generate *RNF8* knockout (*RNF8-KO*) cell lines. To disrupt the human *RNF8* gene, we induced two DSBs in exon 3, screened clones for deletion between the two DSBs by PCR, and then for loss of RNF8 protein using a commercial antibody raised against a portion of RNF8 that is encoded downstream of the DSBs. From this procedure, we isolated two independent *RNF8-KO* cell lines (clones A and B, Figure 3E). We found that similar to mESCs, loss of RNF8 lead to a significant reduction in ALT-EJ as measured by EJ2-GFP in both *RNF8-KO* HEK293 cell lines (2.2- and 2.8-fold for *RNF8*-*KO* clones A and B, respectively) (Figure 3E). We also found that expressing human RNF8 in these *RNF8-KO* cell lines caused a significant increase in the frequency of ALT-EJ (Figure 3E, 1.3-fold for clone A, 1.4-fold for clone B), although RNF8 complementation did not restore ALT-EJ to the frequency of the WT HEK293 cells. This latter finding likely reflects a limitation of complementation experiments to match the conditions of the WT HEK293 cells. Nonetheless, we observed similar results with two independent *RNF8-KO* HEK293 cell lines (Figure 3E). Altogether, these findings support the conclusion that RNF8 promotes ALT-EJ not only in mESCs, but also in HEK293 human cells.

### RNF8 suppresses deletion rearrangement junctions that are hallmarks of C-NHEJ

**Figure 4.**
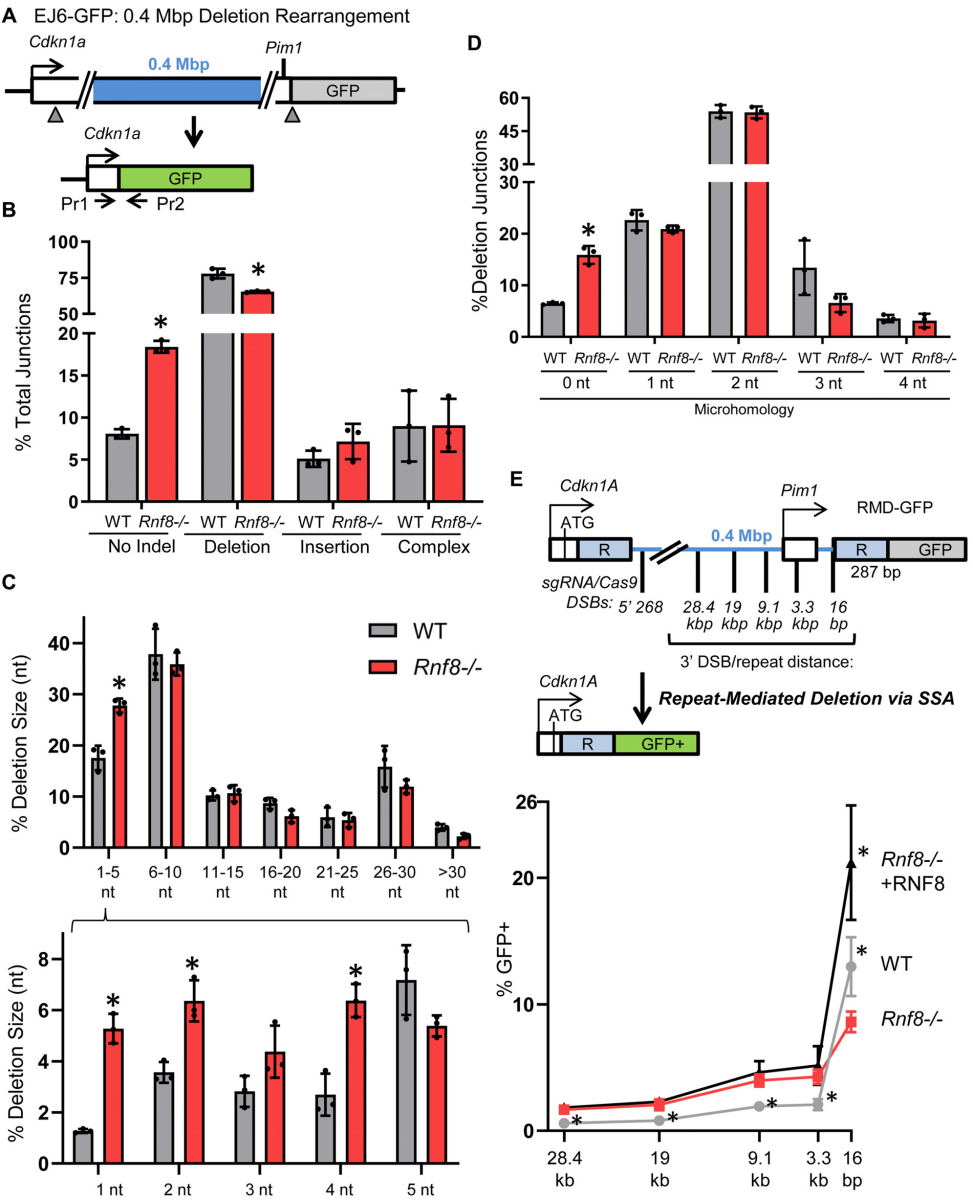
RNF8 suppresses deletion rearrangement junctions that are hallmarks of C-NHEJ, and promotes repeat-mediated deletions with short DSB/repeat distances. (**A**) Shown is a diagram of the EJ6-GFP assay for a 0.4 Mbp deletion rearrangement, as in Figure 1A, but also depicting primers for amplicon deep sequencing of the deletion rearrangement junction. GFP+ cells from the EJ6-GFP deletion rearrangement assay for WT and *Rnf8-/-* mESCs were isolated by cell sorting, and the rearrangements were analyzed by amplicon deep sequencing. (**B**) Shown are the frequencies of four categories of rearrangement junctions: no indel, deletion, insertion and complex. Each data point within the bar represents the frequency from an independent experiment (i.e. a transfection that was sorted for GFP+ cells, which were examined by amplicon sequencing). *N* = 3. Error bars represent SD. (*) *P* ≤ 0.003 for WT versus *Rnf8-/-* mESCs using unpaired multiple *t*-tests with the Holm–Sidak correction. (**C**) RNF8 suppresses very short (1–4 nt) deletion mutations. Shown are two graphs with the frequency of deletion sizes (nt) in WT versus *Rnf8-/-* mESCs, for the deletions shown in (B). The top graph shows deletions placed in 5-nt bins (up to >30-nt), whereas the bottom graph shows the individual deletion sizes between 1 and 5 nt. *N* = 3. Error bars represent SD. (*) *P* ≤ 0.021 for WT versus *Rnf8-/-* mESCs using unpaired multiple *t*-tests with the Holm–Sidak. (**D**) RNF8 suppresses deletion mutations that lack microhomology. From the experiments in (B), sequences with read frequencies of ≥ 0.5% total deletion reads were examined for microhomology usage. Shown is the frequency of microhomology length (0–4 nt) for these deletions. *N* = 3. Error bars represent SD. (*) *P* ≤ 0.007 for WT versus *Rnf8-/-* mESCs frequency, using unpaired multiple *t*-tests with the Holm–Sidak correction. (**E**) RNF8 promotes RMDs if the 3′ DSB/repeat distance is 16 bp, but is dispensable for RMDs with ≥3.3 kb 3′ DSB/repeat distances. Shown is a diagram of the RMD reporter. This reporter uses a GFP cassette fused to a 287 bp repeat that is integrated into the *Pim1* locus, and located 0.4 Mbp downstream of the *Cdkn1A* promoter that is also fused to a 287 bp repeat. Two DSBs are induced in the RMD reporter: one is targeted 268 bp downstream from the 5′ repeat, and the second DSB is targeted upstream of the 3′ repeat at varying distances (16 bp to 28.4 kbp). RMD events that fuse GFP downstream of the *Cdkn1A* promoter, restore GFP+. Also shown are the GFP+ frequencies for WT, *Rnf8-/-* mESCs and *Rnf8-/-* mESCs transfected with 3×F-RNF8 in the RMD reporter. *N* = 9. Error bars represent SD. (*) *P* < 0.001 for unpaired multiple *t*-tests for each 3′ DSB using the Holm–Sidak correction.

The above findings indicate that RNF8 promotes ALT-EJ and inhibits C-NHEJ, but these assays test a few individual EJ outcomes. Thus, we next examined the influence of RNF8 on a broader spectrum of EJ events, using amplicon deep sequencing analysis. For this, we used the EJ6-GFP reporter described above to examine deletion rearrangement junctions. However, in these experiments we did not express TREX2, and hence examined EJ junctions of Cas9-induced DSBs alone, without also inducing degradation of 3′ ssDNA via TREX2. We expressed Cas9 and the sgRNAs to induce the *Cdkn1a*-GFP deletion rearrangement in both WT and *Rnf8-/-* mESCs, isolated GFP+ cells by flow cytometry sorting and amplified the rearrangement junction, which we analyzed by deep sequencing (Figure 4A). We performed this analysis with three independent transfections per cell line, and the results of these replicates were used to determine the mean and standard deviation for the frequency of distinct junction types.

We first organized the sequences by junction type: no indel, deletion, insertion or complex reads (Figure 4B). No indel junctions refer to precise joining events between the distal DSB ends. In a prior study with this assay, the no indel junction type was shown to be absolutely dependent on C-NHEJ (i.e. KU70, XLF and XRCC4) (57). Deletions refer to joining events between the distal DSB ends that also show a loss of nucleotides at the rearrangement junction. Insertions involve an addition of nucleotides at the junction. The final category is complex junctions, which refer to deletions that also have inserted nucleotides. From the analysis of these categories, we found that *Rnf8-/-* mESCs showed a significant increase in no indel junctions (2.3-fold), a modest but significant reduction in deletion junctions (1.2-fold), and no significant difference in insertions or complex junctions, each as compared to WT mESCs.

We also examined two features of the deletion mutations: deletion size and microhomology usage. We examined the size of all deletions binned into groups of 5-nt, and found that *Rnf8-/-* mESCs showed significant increase (1.6-fold) in very short deletions (1–5 nt), compared to WT (Figure 4C). Furthermore, by examining this 1–5 nt category in more detail, *Rnf8-/-* mESCs showed significantly higher frequencies of 1, 2 and 4-nt deletions, as compared to WT (Figure 4C). We also examined microhomology usage for sequences with read frequencies of ≥0.5% total deletion reads (see ‘Materials and Methods’ section). From this analysis, we found that *Rnf8-/-* mESCs had a substantial increase (2.5-fold) in events without microhomology (0 nt) as compared to WT mESCs (Figure 4D). In contrast, we did not observe an increase in events with microhomology (1, 2, 3 or 4-nt) in *Rnf8-/-* versus WT (Figure 4D). In summary, loss of RNF8 causes an increase in junctions without indels, very short (1–4 nt) deletions and deletions without microhomology. Each of these junction types are hallmarks of C-NHEJ, as they reflect EJ with limited end processing and/or without use of microhomology (54–57). Accordingly, these findings indicate that RNF8 suppresses deletion rearrangements mediated by C-NHEJ.

### RNF8 is important for repeat-mediated deletions with short DSB/repeat distances

Since RNF8 appears to suppress C-NHEJ, and promote ALT-EJ events that require limited DSB end resection (i.e. the 4-μHOM Embed event), we wondered whether RNF8 affects repair events requiring longer end resection. To test this, we examined repeat-mediated deletions (RMDs) via single strand annealing (SSA) that require varying amounts of DSB end resection. In this assay (RMD-GFP), two identical 287 bp sequences (i.e. two repeat sequences) are separated by 0.4 Mbp on chromosome 17 (Figure 4E). The 5′ repeat is within the endogenous *Cdkn1a* locus, whereas the 3′ repeat has been inserted into the *Pim1* locus, and fused to the GFP coding sequence. An RMD between the repeats creates a *Cdkn1a-GFP* fusion gene, which can be detected as GFP+ cells. To induce the RMD, Cas9 is used to target two DSBs: one is targeted 268 bp downstream from the 5′ repeat, and the second DSB is targeted upstream of the 3′ repeat at varying distances (16 bp to 28.4 kbp). We examined these RMDs in *Rnf8-/-* mESCs, which we compared to WT cells and the mutant cell line co-transfected with the 3×F-RNF8 expression vector. We found that loss of RNF8 caused a reduction in the RMD event using the shortest 3′ DSB/repeat distance (16 bp), compared to WT (1.5-fold), and the complemented condition (2.5-fold, Figure 4E). In contrast, loss of RNF8 did not cause a decrease in the RMDs with the longer DSB/repeat distances (3.3–28.4 kbp). These findings indicate that RNF8 is specifically important for SSA events requiring relatively limited end resection.

### The role of RNF8 on ALT-EJ is dependent on KU70

**Figure 5.**
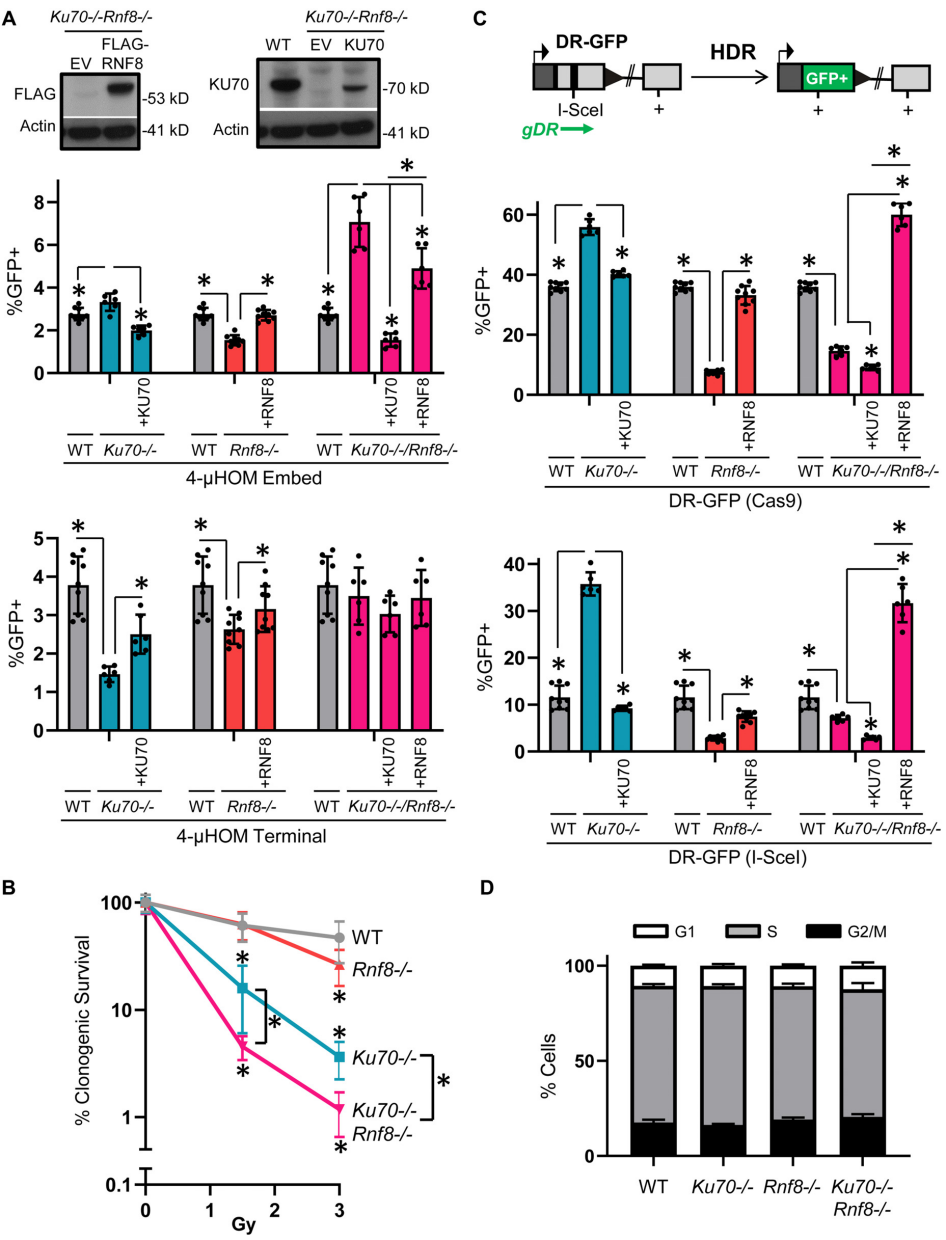
The role of RNF8 on ALT-EJ is dependent on KU70, whereas RNF8 promotes HDR independently of KU70. (**A**) RNF8 has a KU dependent role in ALT-EJ. A double mutant *Ku70-/-Rnf8-/-* mESC line was generated to examine the influence of RNF8 on DSB repair in the absence of KU70. Shown are the GFP+ frequencies for 4-μHOM Embed (top) and 4-μHOM Terminal (bottom) for WT, *Ku70-/-*transfected with and without KU70, and *Ku70-/-Rnf8-/-* without complementation vector, or transfected with KU70 or 3xF-RNF8. *N* = 6, except *N* = 9 for WT and *Rnf8-/-*. Error bars represent SD. (*) *P* ≤ 0.039 for unpaired multiple *t*-tests with the Holm–Sidak correction. The GFP+ frequencies from Figure 3C for the 4-μHOM assay for WT and *Rnf8-/-* are shown for comparison. Shown are immunoblot signals for Flag and Actin control in *Ku70-/-Rnf8-/-* transfected with and without 3×F-RNF8, and for KU70 and Actin control in WT and *Ku70-/-Rnf8-/-* transfected with and without KU70. (**B**) The influence of KU70 and RNF8 on radioresistance is not epistatic. Shown is clonogenic survival of WT, *Ku70-/-*, *Rnf8-/-* and *Ku70-/-Rnf8-/-* following 1.5 or 3 Gy of radiation exposure, normalized to untreated (0 Gy = 100%). *N* = 6. Error bars represent SD. (*) *P* ≥ 0.045 for WT versus each of the mutant cell lines and *Ku70-/-* versus *Ku70-/-Rnf8-/-* using unpaired multiple *t*-tests for each treatment dose, with the Holm–Sidak correction. (**C**) RNF8 promotes HDR independently of KU70. Shown is a diagram of the DR-GFP reporter for HDR, which can be used to measure repair of DSBs induced by either sgRNA/Cas9 or I-SceI. Shown are the GFP+ frequencies for DR-GFP induced by Cas9 (top) and I-SceI (bottom) in WT mESCs, *Rnf8-/-* mESCs transfected with and without the 3×F-RNF8 expression vector, *Ku70-/-* mESCs transfected with and without the KU70 expression vector, *Ku70-/-Rnf8-/-* mESCs without complementation vector, and with the KU70 and 3×F-RNF8 expression vectors. *N* = 6, except *N* = 9 for WT and *Rnf8-/-*. Error bars represent SD. (*) *P* ≤ 0.001 for unpaired multiple *t*-tests with the Holm–Sidak correction. (**D**) Loss of KU70 and/or RNF8 does not have obvious effects on cell-cycle phase. Based on BrdU labeling and DNA counterstain (propidium iodide), shown is the frequency of cells in G1, S and G2/M, for WT, *Ku70-/-*, *Rnf8-/-* and *Ku70-/-Rnf8-/-* mESCs. *N* = 3. Error bars represent SD.

We next considered possible roles of RNF8 during ALT-EJ, such as inhibition of KU (i.e. the KU70/KU80 heterodimer), which binds DSB ends to mediate C-NHEJ (4,10). Namely, a key step of ALT-EJ is likely the displacement of KU from DSB ends to enable limited end resection and/or binding of downstream ALT-EJ factors (e.g. POLQ). Accordingly, we posited that the role of RNF8 during EJ might be dependent upon KU. A corollary of this hypothesis is that KU loss will suppress the requirement of RNF8 for ALT-EJ. To test this notion, we generated a *Ku70-/-Rnf8-/-* mESC line from the *Rnf8-/-* mESC line, and then integrated the 4-μHOM reporter (Figure 5A). We then evaluated the 4-μHOM Embed assay for ALT-EJ in the *Ku70-/-Rnf8-/-* mESC line, compared to WT, as well as the mutant line with expression of the individual complementation vectors for KU70 and 3×F-RNF8 (Figure 5A). For comparison, we also included analysis of the single mutants, and found that loss of KU70 causes a significant increase in the 4-μHOM Embed EJ event, whereas loss of RNF8 causes a reduction in this event, each compared both to WT and the complemented condition (Figures 3C and 5A). In cells deficient in both factors (*Ku70-/-Rnf8-/-*), we found a striking increase in the 4-μHOM Embed EJ event, compared to both WT and the single mutants (e.g. 2.6-fold higher than WT, Figure 5A). The KU70 expression vector caused a marked reduction in this EJ event in the *Ku70-/-Rnf8-/-* cells (Figure 5A). Furthermore, the 3×F-RNF8 expression vector caused a significant reduction in this EJ event in the *Ku70-/-Rnf8-/-* cells, which is in marked contrast to the increase in such EJ caused by 3×F-RNF8 expression in the single mutant (i.e. *Rnf8-/*, Figures 3C and 5A). These findings indicate that in the absence of KU70, RNF8 no longer promotes ALT-EJ, but rather suppresses this event. Thus, the role of RNF8 in promoting ALT-EJ is dependent on KU.

For comparison, we also performed analysis of the 4-μHOM Terminal EJ event, which is induced by sgRNAs that target the edge of the microhomology (see Figure 2A). As described above, RNF8 has a lesser role on this EJ event with terminal microhomology, compared to embedded microhomology (Figure 3C). We found that *Ku70-/-* cells showed a modest, but significant reduction of this EJ event, compared to either WT or the complemented condition (Figure 5A). Finally, combined loss of both factors in the *Ku70-/-Rnf8-/-* cells showed no obvious difference in the 4-μHOM Terminal EJ event, compared to WT or the complemented conditions (Figure 5A). These findings indicate that RNF8 and KU70 have the greatest influence on EJ with microhomology that is embedded from the edge of the DSB, compared to EJ with terminal microhomology.

### RNF8 promotes HDR independently of KU70

Because the role of RNF8 in ALT-EJ is KU dependent, we next tested whether RNF8 has other roles in chromosomal break repair that are independent of KU. To begin with, we examined sensitivity to IR. Specifically, we examined the influence of 1.5 and 3 Gy IR treatment on the clonogenic survival of WT, *Ku70-/-*, *Rnf8-/-* and *Ku70-/-Rnf8-/-* mESCs. We found that loss of RNF8 caused a substantial reduction in clonogenic survival at 3 Gy IR treatment, as compared to WT (Figure 5B, 1.8-fold). We also found that loss of KU70 lead to a substantial reduction in clonogenic survival at both IR doses, as compared to WT (Figure 5B, 3.8- and 12.9-fold, at 1.5 and 3 Gy, respectively). Moreover, we found that combined loss of both factors (*Ku70-/-Rnf8-/-* mESCs) showed substantial reduction in clonogenic survival, compared to the *Ku70-/-* single mutant (Figure 5B, 3.5- and 3.1-fold, for 1.5 and 3 Gy, respectively). This finding indicates that RNF8 is important for IR-resistance in both KU70-proficient and KU70-deficient cells.

Given that RNF8 has a role in IR-resistance in KU70-deficient cells, but its influence on ALT-EJ is suppressed by loss of KU70, we considered that RNF8 has a role in other aspects of chromosomal break repair. In particular, we posited that RNF8 is important for HDR and that this role is independent of KU. To test this hypothesis, we used the DR-GFP reporter that measures a gene conversion event (Figure 5C) (71). This reporter can be used to examine repair of DSBs induced by either Cas9, or the meganuclease I-SceI (61). Thus, we measured the influence of RNF8 and KU on HDR induced by both Cas9 and I-SceI, using the mutant mESC lines and complementation vectors described above. We found that loss of RNF8 lead to a marked decrease in HDR, as compared to WT, which was rescued with the 3×F-RNF8 expression vector (Figure 5C). In contrast, we found that loss of KU70 caused an increase in HDR, compared to both WT and the complemented condition, albeit with a greater fold effect for HDR induced with I-SceI versus Cas9 (Figure 5C, 3.1-fold versus 1.6-fold, respectively). Importantly, we found that combined loss of KU70 and RNF8 (*Ku70-/-Rnf8-/-* mESCs) caused a substantial reduction in HDR as compared to WT (Figure 5C). Furthermore, the RNF8 expression vector caused a marked increase in HDR in the *Ku70-/-Rnf8-/-* mESCs, whereas the KU70 expression vector caused a reduction in HDR in these cells (Figure 5C). Altogether, these findings indicate that RNF8 promotes HDR independently of KU.

We also performed a set of controls with these cell lines. By using multiple DSB reporters to examine distinct repair outcomes and normalizing repair values to transfection efficiency, we have controlled for non-specific effects on Cas9 expression and activity, since any such effects on Cas9 should affect all outcomes equivalently. Nevertheless, as an additional control, we examined expression of Cas9 in multiple conditions, by performing Flag immunoblot analysis, since the Cas9 we use in our experiments has a Flag immunotag. We transfected WT, *Ku70-/-*, *Rnf8-/-* and *Ku70-/-Rnf8-/-* mESCs with the sgRNA/Cas9 expression vectors and a puromycin-resistance expression plasmid, and treated cells with puromycin to enrich for transfected cells prior to protein extraction. We also performed this analysis for the siRNA treatments shown in Figure 2, namely WT cells treated with siCTRL, siCtIP, siBRCA1 and siNBS1. From this analysis, we found no significant effects on Cas9 expression among the WT, *Ku70-/-*, *Rnf8-/-* and *Ku70-/-Rnf8-/-* mESCs or the siRNA treated WT cells (Supplementary Figure S3A).

As another control for Cas9 activity, we examined mutagenic loss of the Cas9 target site in the DR-GFP reporter. Specifically, following expression of the sgRNA/Cas9 to induce the DSB at the I-SceI recognition site in DR-GFP, we quantified loss of the I-SceI site, using PCR and I-SceI restriction digestion analysis (Supplementary Figure S3B). We chose this approach because this assay has been validated in prior reports (63,67). Furthermore, loss of the I-SceI site in DR-GFP can occur by several repair pathways (i.e. ALT-EJ, mutagenic C-NHEJ, HDR and SSA) (63,67). Thus, loss of the I-SceI site in DR-GFP is not biased by any particular repair outcome, except for not being able to detect precise EJ that restores the I-SceI site. We performed this assay in WT, *Ku70-/-*, *Rnf8-/-* and *Ku70-/-Rnf8-/-* mESCs with the DR-GFP reporter, described above, since these cell types are the primary focus of our study. From this analysis, we found that WT and *Rnf8-/-* mESCs did not show statistical differences for loss of the I-SceI site (Supplementary Figure S3B and C). For the *Ku70-/-* and *Ku70-/-Rnf8-/-* mESCs, we observed a significant increase in the frequency of loss of the I-SceI site, compared to WT (Supplementary Figure S3B and C). These latter findings are consistent with prior studies of effects of KU on I-SceI site loss with DR-GFP (72), and is likely due to the role of KU in suppressing all of the repair events that cause loss of the I-SceI site (8,73).

As another control, since HDR is cell cycle regulated (74,75), we examined cell-cycle profiles of the WT, *Ku70-/-*, *Rnf8-/-* and *Ku70-/-Rnf8-/-* mESC lines using BrdU labeling and propidium iodide counterstain (Figure 5D). We also performed this experiment after transfecting cells with an sgRNA/Cas9 expression vector, and a puromycin-resistance expression vector that was used to enrich for transfected cells (Supplementary Figure S3D). For the latter, we used the EJ7-GFP cell lines, and an sgRNA targeting one DSB in this reporter, since the single DSB does not induce GFP+ cells. We needed to avoid GFP expression in this experiment, because it would complicate the BrdU analysis, which uses the FITC fluorochrome. With both approaches to cell cycle analysis, we found no significant effects on cell cycle profiles among the WT, *Ku70-/-*, *Rnf8-/-*, and *Ku70-/-Rnf8-/-* mESCs (Figure 5D and Supplementary Figure S3D). Altogether, these controls support the conclusion that RNF8 affects distinct repair outcomes in a manner that cannot be readily explained by changes to Cas9 expression/activity, or cell-cycle phase.

### RNF8 promotes RAD51 foci formation, the role of RNF8 in HDR can be partially suppressed with a fusion protein of the RNF8-FHA domain with PALB2 and the RNF8 RING domain is important for repair

**Figure 6.**
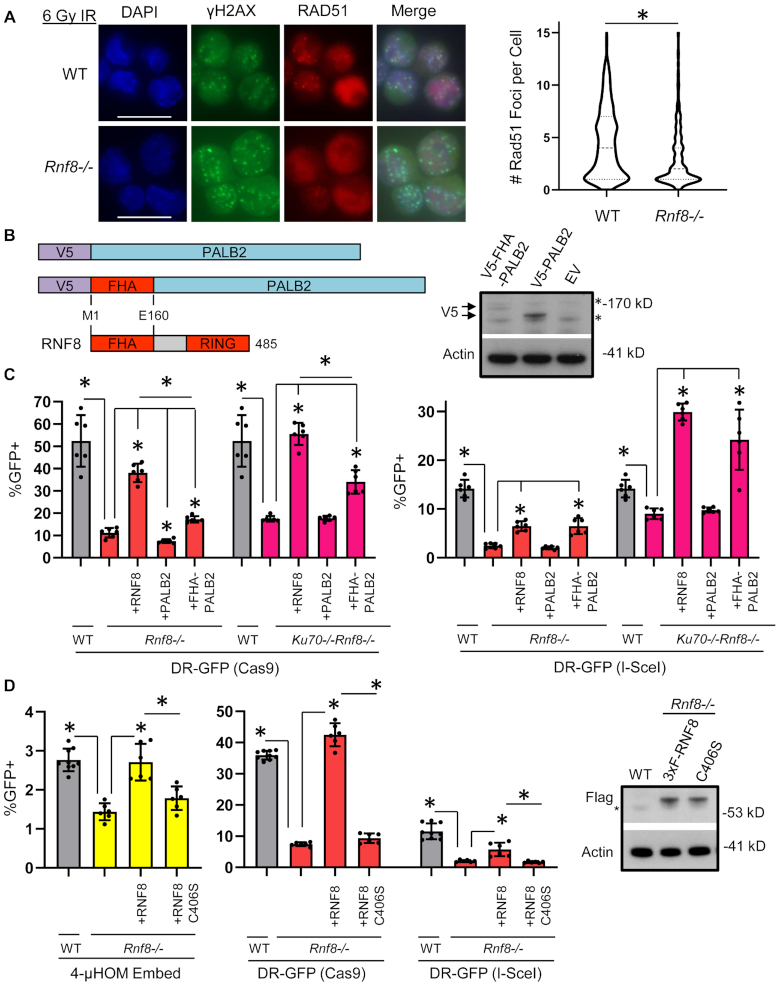
RNF8 promotes RAD51 foci, the role of RNF8 in HDR can be partially suppressed with a fusion protein of the RNF8-FHA domain with PALB2 and the RNF8 RING domain is important for repair. (**A**) RAD51 foci are reduced in *Rnf8-/-* mESC. Shown are representative fluorescent images of WT and *Rnf8-/-* mESCs treated with 6 Gy, recovered for 4 h and stained with DAPI and antibodies targeting γH2AX and RAD51. Scale bar = 20 μm. Also shown are violin plots depicting the number of RAD51 foci per nucleus that contained ≥5 γH2AX foci in WT and *Rnf8-/-* mESC. *N* > 350. (*) *P* < 0.001 for WT versus *Rnf8-/-* measuring RAD51 foci using the Mann–Whitney test. (**B**) Shown are diagrams (not to scale) of V5-PALB2 and V5-FHA-PALB2, along with the segment of RNF8 used for the FHA domain fusion. V5 is an immunotag. (**C**) Expressing the V5-FHA-PALB2 fusion protein depicted in (B) promotes HDR in RNF8-deficient cells. Shown are the GFP+ frequencies for DR-GFP induced by Cas9 (left) and I-SceI (right) in WT mESCs, and *Rnf8-/-* and *Ku70-/-Rnf8-/-* mESCs transfected without complementation vector, or with expression vectors for RNF8, PALB2, or FHA-PALB2. *N* = 6. Error bars represent SD. (*) *P* ≤ 0.003 for unpaired multiple *t*-tests with the Holm–Sidak correction. Shown are V5 and Actin control immunoblot signals in *Rnf8-/-* mESCs with and without transfection of V5-FHA-PALB2 and V5-PALB2. Arrows represent V5-FHA-PALB2 (top arrow) and V5-PALB2 (bottom arrow). (**D**) The RNF8 RING domain is required to promote ALT-EJ and HDR. Shown are the GFP+ frequencies for 4-μHOM Embed, and DR-GFP induced with both Cas9 (left) and I-SceI (right) in WT mESCs, and *Rnf8-/-* mESCs transfected without complementation vector, or with expression vectors for 3×F-RNF8 (WT) or C406S. *N* = 6. WT mESC values from Figures 3C and 5C are shown for comparison (*N* = 9). Error bars represent SD. (*) *P* ≤ 0.019 for unpaired multiple *t*-tests with the Holm–Sidak correction.

Since the role of RNF8 in HDR is independent of KU70, we posited that RNF8 may promote steps of HDR after displacement/inhibition of KU. Consistent with this notion, recent studies have implicated RNF8 in promoting the function of PALB2, which is a critical HDR factor that recruits BRCA2 to DNA damage, and is required for subsequent recruitment of the RAD51 recombinase (35–36,76). In particular, RNF8 is important for recruitment of another E3 ubiquitin ligase, RNF168 to DNA damage, which appears to promote PALB2 function (35–36,76). Accordingly, we posited that RNF8 is important for PALB2 function, and therefore also is important for RAD51 recruitment. Thus, we first tested whether RNF8 is important for recruitment of RAD51 to DNA damage, by examining RAD51 foci formation following IR treatment (Figure 6A). From this analysis, we found that RNF8 loss causes a marked reduction in RAD51 foci, compared to WT cells (Figure 6A). Thus, RNF8 is important for recruitment of RAD51 to DNA damage.

To examine PALB2 function, we posited that fusing the RNF8-FHA domain to PALB2 may rescue the requirement of RNF8 for HDR (FHA-PALB2, Figure 6B). This hypothesis is based on a recent report that a similar fusion protein could rescue RAD51 foci in cells that are deficient for one allele of *Brca1*, and also lack *Rnf168* (36). The RNF8-FHA domain binds to sites of DNA damage via interaction with MDC1, which is recruited to DNA damage through an interaction with γH2AX (20–22,29). Thus, we tested whether an expression vector for FHA-PALB2 may promote HDR in RNF8-deficient cells, using the DR-GFP assay. We expressed V5-immunotagged FHA-PALB2, and V5-PALB2, in both *Rnf8-/-* and *Ku70-/-Rnf8-/-* mESCs, which we compared to empty expression vector and the 3×F-RNF8 expression vector. We confirmed expression of these PALB2 proteins using the V5-immunotag (Figure 6B). From these experiments, we found that expression of V5-FHA-PALB2, but not V5-PALB2, caused a significant increase in HDR in both *Rnf8-/-* and *Ku70-/-Rnf8-/-* mESCs (Figure 6C). For repair of DSBs induced by I-SceI, FHA-PALB2 rescued HDR to a similar degree as expression of RNF8, whereas for Cas9 the rescue was less than for RNF8 (Figure 6C). These findings indicate that the FHA-PALB2 fusion protein partially suppresses the HDR defect in RNF8-deficient cells.

While the roles of RNF8 during ALT-EJ and HDR appear distinct, regarding the requirement for KU, we posited that both functions would be dependent upon its RING domain, which is critical for its E3 ubiquitin ligase activity (21,24). Thus, we examined a mutant of RNF8 with a conserved cysteine within the RING domain (C403 in human, and C406 in mouse), changed to serine (i.e. mouse C406S) (21,24). We compared the effect of expressing 3×F-RNF8 WT versus C406S in *Rnf8-/-* mESCs on the frequency of ALT-EJ (4-μHOM Embed) and HDR (DR-GFP, both with Cas9 and I-SceI). We found that expression of 3×F-RNF8 WT, but not C406S, caused a significant increase in these repair events (Figure 6D, expression confirmed with Flag immunoblot). These findings indicate that the RNF8 RING domain is necessary to promote both ALT-EJ and HDR.

### 53BP1 does not have a substantial effect on either EJ without indels or ALT-EJ

**Figure 7.**
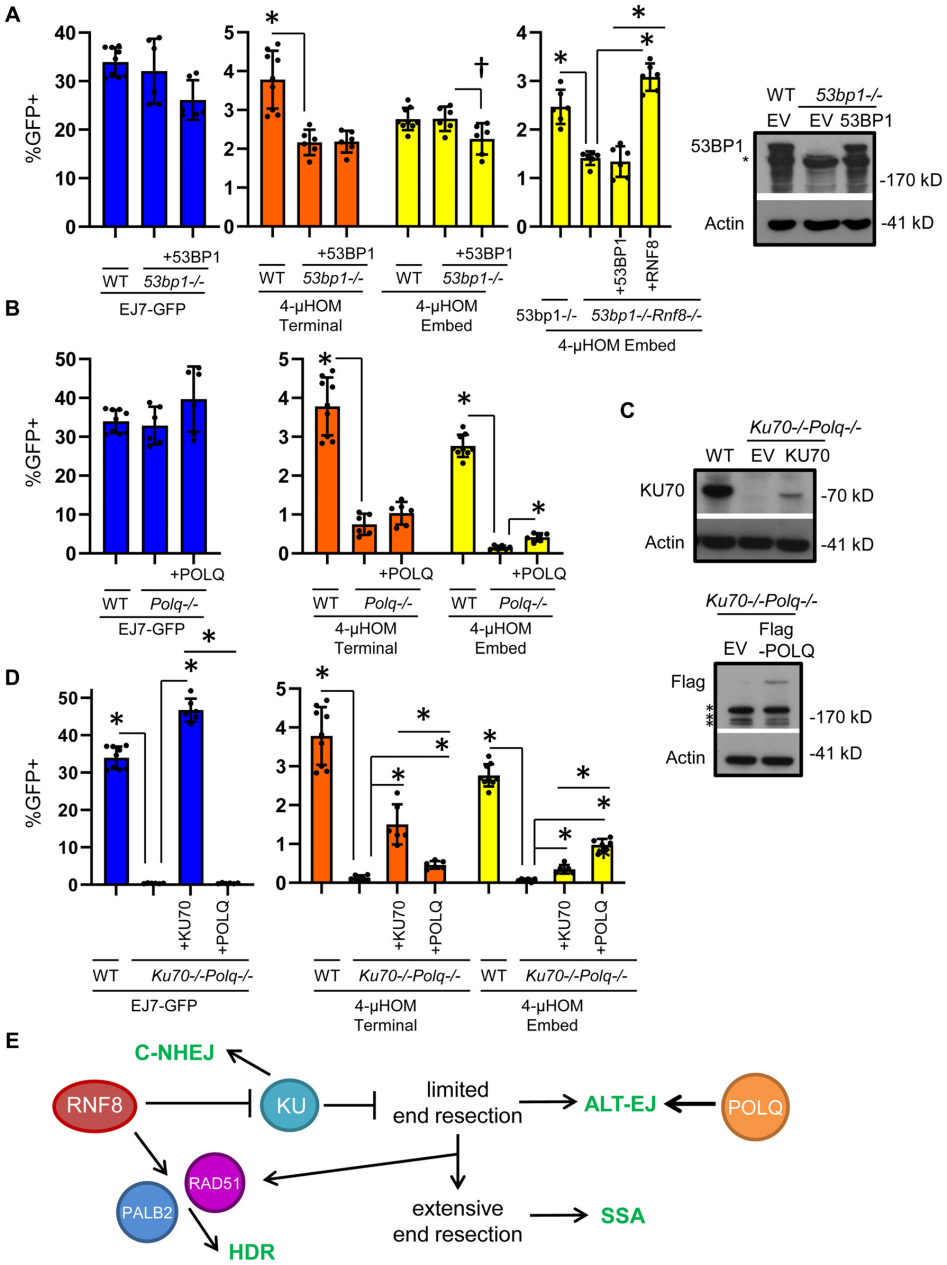
53BP1 does not have a substantial effect on either EJ without indels or ALT-EJ, whereas POLQ promotes ALT-EJ independently of KU. (**A**) Influence of 53BP1 on distinct EJ events. Shown are the GFP+ frequencies for several chromosomal reporter assays (EJ7-GFP, 4-μHOM Terminal and 4-μHOM Embed) in WT mESCs, *53bp1-/-* mESCs and *53bp1-/-* mESCs transfected with an expression vector for human 53BP1. Also shown are GFP+ frequencies for 4-μHOM Embed in *53bp1-/-Rnf8-/-* mESCs transfected with and without 53BP1 and RNF8 (3×F-RNF8) expression vectors, and compared to *53bp1-/-* mESCs. WT values for EJ7-GFP and 4-μHOM are from Figures 1C and 3C, respectively, and are shown for comparison. *N* = 6, except *N* = 9 for WT. Error bars represent SD. (*) *P* ≤ 0.049 using unpaired multiple *t*-tests with the Holm–Sidak correction. Also shown is an immunoblot staining for 53BP1 and Actin control in WT and *53bp1-/-* mESCs transfected with and without 53BP1. (*) represent non-specific bands. (**B**) Influence of POLQ on distinct EJ events. Shown are the GFP+ frequencies for several chromosomal reporter assays (EJ7-GFP, 4-μHOM Terminal and 4-μHOM Embed) in WT mESCs, *Polq-/-* mESCs and *Polq-/-* mESCs transfected with human Flag-tagged POLQ (POLQ). WT values for EJ7-GFP and 4-μHOM are from Figures 1C and 3C, respectively, and are shown for comparison. *N* = 6, except *N* = 9 for WT. Error bars represent SD. (*) *P* < 0.001 for unpaired multiple *t*-tests with the Holm–Sidak correction. (**C**) Immunoblot analysis of KU70, Flag-POLQ and Actin control. A double mutant *Ku70-/-Polq-/-* mESC line was generated to examine the influence of POLQ and KU70 on EJ independently of the other factor. Shown are KU70 immunoblot signals for WT, and *Ku70-/-Polq-/-* mESCs transfected with and without KU70. Also shown are Flag and Actin control immunoblot signals for *Ku70-/-Polq-/-* mESCs transfected with and without Flag-tagged POLQ. (*) indicates a non-specific band. (**D**) POLQ and KU70 independently mediate distinct EJ events. Shown are the GFP+ frequencies for several chromosomal reporter assays (EJ7-GFP, 4-μHOM Terminal and 4-μHOM Embed) in WT mESCs, and *Ku70-/-Polq-/-* mESCs without complementation vector, or with expression vectors for KU70 or POLQ. WT values for EJ7-GFP, and 4-μHOM are from Figures 1C and 3C, respectively, and shown for comparison. *N* = 6, except *N* = 9 for WT. Error bars represent SD. (*) *P* < 0.001 for unpaired multiple *t*-tests with the Holm–Sidak correction. (**E**) Shown is a model for the influence of RNF8 on chromosomal break repair outcomes.

Since RNF8 is required for the recruitment of a key DDR factor, 53BP1, to sites of DNA damage (20–22), we wondered if 53BP1 has similar effects on EJ as RNF8. We also examined 53BP1, because this factor is critical for several EJ events, such as class switch recombination and fusion of deprotected telomeres (77), but its influence on repair of Cas9-induced DSBs has been unclear. To examine the influence of 53BP1 on EJ of Cas9-induced DSBs, we integrated the EJ7-GFP and the 4-μHOM assays into a previously described *53bp1-/-* mESC line (57). We compared *53bp1-/-* mESCs versus WT, and the mutant cells co-transfected with an expression vector for human 53BP1, which we confirmed by immunoblotting (Figure 7A). For EJ7-GFP (EJ without indels), 53BP1 loss had no effect, compared to either WT or the complemented condition (Figure 7A). For the 4-μHOM Embed assay, *53bp1-/-* was not distinct from WT, although including the 53BP1 expression vector in mutant cell line caused a modest decrease (Figure 7A, 1.2-fold, significant only without adjustment for multiple comparisons). For the 4-μHOM Terminal assay, the frequency of this EJ event was lower in *53bp1-/-* versus WT (1.75-fold), but was not affected by the complementation vector (Figure 7A). While these findings may indicate that 53BP1 is important for EJ using 4-nt of terminal microhomology, the lack of complementation with the 53BP1 expression vector raises the possibility that the reduction in this EJ event may not be due to loss of 53BP1. In any case, 53BP1 has no clear effect on the EJ7-GFP assay for EJ without indels, which is distinct both from the inhibitory role of RNF8 and the mediator role of KU70, shown above. Furthermore, 53BP1 appears dispensable for the 4-μHOM Embed event, which is distinct from the mediator role of RNF8.

Based on these findings, we posited that the role of RNF8 in ALT-EJ may be independent of 53BP1. To test this, we generated a *53bp1*-/-*Rnf8*-/- from the *53bp1*-/- mESC line with the 4-μHOM reporter (Supplementary Figure S1B), and then performed the 4-μHOM Embed assay to examine ALT-EJ. We found that loss of RNF8 in *53bp1*-/- mESCs caused a significant decrease in ALT-EJ (1.7-fold) (Figure 7A), which was similar to the effect of RNF8 loss in WT cells, described above (1.8-fold, Figure 3C). We also found that the 53BP1 expression vector had no effect on the frequency of ALT-EJ in the *53bp1*-/-*Rnf8*-/- mESCs, similar to the finding with the *53bp1*-/- single mutant (Figure 7A). Finally, the RNF8 expression vector caused a significant increase in ALT-EJ in the *53bp1*-/-*Rnf8*-/- mESCs (Figure 7A, 2.2-fold), which is similar to the finding with the *Rnf8*-/- single mutant (Figure 3C). Thus, the influence of RNF8 on mediating ALT-EJ cannot readily be attributed to its function to promote 53BP1 recruitment to DNA damage.

### POLQ promotes ALT-EJ independently of KU70

Finally, we sought to compare the influence of RNF8 on EJ with that of POLQ, which promotes microhomology annealing and primer extension during ALT-EJ (78). In a prior study from our laboratory, we examined the influence of POLQ on multiple EJ events, using a *Polq-/-* mESC line (54,62). We repeated these studies in the *Polq-/-* line with several of the EJ reporters described above: EJ7-GFP (no indel), 4-μHOM Embed and 4-μHOM Terminal (Figure 7B). From this analysis, we found that the 4-μHOM Embed, and 4-μHOM Terminal events markedly reduced in *Polq-/-* mESCs versus WT (Figure 7B, 5.0- and 18.8-fold for Terminal and Embed, respectively). We also found that co-transfecting an expression vector for Flag-immunotagged human POLQ caused a significant increase in 4-μHOM Embed EJ in *Polq-/-* mESCs, although the frequencies were not restored to WT (Figure 7B). In contrast, we found that that *Polq-/-* mESCs showed no significant difference in EJ without indels (EJ7-GFP), compared to either WT or the complemented condition (Figure 7B).

We next examined the influence of POLQ on EJ in the absence of KU70, using a *Polq-/-Ku70-/-* double-mutant mESC line that we generated from the *Polq-/-* line (Figure 7C). We then integrated several EJ reporters, and examined the frequency of these EJ events, as compared to WT, as well as the double-mutant line co-transfected with the relevant complementation vector (KU70 or Flag-tagged POLQ). We confirmed expression of both Flag-POLQ and KU70 from the respective complementation vectors by immunoblotting in the *Polq-/-Ku70-/-* mESCs (Figure 7C). From these experiments, we found that the *Polq-/-Ku70-/-* mESCs showed a marked loss in all EJ events, compared to WT: EJ7-GFP (EJ without indels), 4-μHOM Terminal and 4-μHOM Embed (Figure 7D). We also found that expression of KU70, but not Flag-POLQ, caused a significant increase in the EJ7-GFP event (EJ without indels, Figure 7D). In contrast, for both the 4-μHOM Terminal and 4-μHOM Embed EJ events, expression of either Flag-POLQ or KU70 caused a significant increase in these EJ events (Figure 7D). The latter result is distinct from the influence of KU70 in the presence of POLQ (i.e. the *Ku70-/-* single mutant), in which KU70 suppresses the 4-μHOM Embed EJ event (Figure 5A). Although, in *Polq-/-Ku70-/-* mESCs, KU70 expression had a greater effect on mediating the 4-μHOM Terminal versus 4-μHOM Embed (11.7-fold versus 5.2-fold, respectively). Conversely, Flag-POLQ expression had a greater effect on 4-μHOM Embed versus 4-μHOM Terminal (14.5-fold versus 3.6-fold, respectively). These findings indicate that either POLQ or KU70 are required for each the diverse set of EJ events examined here. Thus, whereas RNF8 appears to be important for ALT-EJ only in KU70-proficient cells, the role of POLQ in ALT-EJ is not dependent on KU70 (Figure 7E).

Based on these findings, we posited that RNF8 may function independently of POLQ during DSB repair. To test this, we attempted to generate a *Rnf8*-/-*Polq*-/- mESC from the *Polq*-/- mESC line, using the same *Rnf8* knockout strategy used for the rest of the study, but were unable to generate this double mutant after screening 197 independent clones (Supplementary Table S2). The failure to generate this double mutant indicates that such a cell line might be inviable, indicating that RNF8 and POLQ may have distinct/non-epistatic roles in genome maintenance. This finding is consistent with the reports that combined loss of POLQ and 53BP1 is synthetic lethal (5,79), because, as mentioned above, 53BP1 recruitment to DSBs is dependent on RNF8 (20–22). Altogether, these findings indicate that the role of RNF8 and POLQ during ALT-EJ and genome maintenance are distinct.

## DISCUSSION

RNF8 is critical for ubiquitin signaling events at DSBs that regulate the recruitment of several DDR factors to sites of DNA damage (20–22,29,80). Nonetheless, the precise role of RNF8 on distinct DSB repair outcomes has been unclear, in part due to a lag in the development of robust approaches to distinguish C-NHEJ versus ALT-EJ. Recent studies of Cas9-induced DSBs, which are largely blunt ended, have demonstrated that C-NHEJ is required for EJ of such DSBs without indel mutations (54–57). We have found that RNF8 is important to suppress this repair event. Indeed, through examining EJ repair junctions via amplicon deep sequencing, the most striking effect of RNF8 loss appears to be an increase in EJ without indel mutations. Conversely, RNF8 is important to promote ALT-EJ events using 4-nt of microhomology that is embedded from the edge of the DSB by 8-nt (i.e. the 4-μHOM Embed assay). RNF8 also promotes an ALT-EJ event measured by a distinct assay (EJ2-GFP), in both mESCs and HEK293 human cells. Altogether, RNF8 appears to both inhibit C-NHEJ and promote ALT-EJ, but these effects may likely be due to a single mechanism. Namely, mechanisms that inhibit C-NHEJ are likely also important to facilitate ALT-EJ, since these repair outcomes are in competition.

A possible role for RNF8 for promoting ALT-EJ/inhibiting C-NHEJ is to facilitate DSB end resection to generate 3′ ssDNA substrates for the microhomology annealing. However, we also found that RNF8 is dispensable for repair events that likely involve extensive end resection (i.e. SSA involving long DSB/repeat distances). These findings with RNF8 are similar to H2AX, which also has been shown to suppress EJ without indels (57,81), is not required for SSA, and indeed has been shown to suppress an SSA event causing a 3 kbp deletion (82). In contrast, CtIP is important to inhibit EJ without indels, promote ALT-EJ (4-μHOM Embed assay), and promote SSA induced by long DSB/repeat distances (59). The other two factors we identified in our RNAi screen, NBS1 and BRCA1, appear to have similar effects as CtIP, in that each of these factors promote ALT-EJ (4-μHOM Embed assay) and promote SSA (BRCA1 and NBS1 promote an SSA event causing a 2.7 kb deletion, and BRCA1 also mediates SSA induced by long DSB/repeat distances) (59,82–83). In summary, RNF8 and H2AX appear specifically important for limited end resection for ALT-EJ, but not extensive resection of several kb to facilitate SSA. Conversely, factors such as CtIP appear important for both limited end resection for ALT-EJ, as well as extensive resection for SSA. Thus, these findings indicate limited end resection for ALT-EJ is mechanistically distinct from extensive resection to facilitate SSA.

We also found that ALT-EJ events requiring limited end resection are distinct from EJ events that use microhomology at the edge of the DSB. Namely, RNF8, CtIP, BRCA1 and NBS1 showed a greater effect in promoting the 4-μHOM Embed versus 4-μHOM Terminal EJ events. Similarly, from our amplicon deep sequencing analysis of rearrangement junctions, we found that RNF8 inhibits short (1–4 nt) deletions, such that these EJ events are also likely mechanistically distinct from ALT-EJ. Finally, while KU70 suppresses the 4-μHOM Embed EJ event, this factor modestly promotes the 4-μHOM Terminal EJ event. As one possibility for these distinctions, use of terminal microhomology could involve disruption of DSB end base pairing without either displacement of the C-NHEJ complex, or end resection *per se*.

In contrast, limited end resection for ALT-EJ likely requires displacement of KU from DSB ends. Consistent with this notion, KU suppresses ALT-EJ measured by the 4-μHOM Embed assay. RNF8 could mediate such KU displacement through direct ubiquitination, as has been shown in several studies (29,50–51). In support of this model, we find that loss of KU70 suppresses the requirement of RNF8 for ALT-EJ. This model is also supported by findings that RNF8 limits KU retention at LASER DNA damage, although this result appears controversial (29,50–51). In addition to directly affecting KU, RNF8 could displace KU through promoting efficient recruitment of NBS1. Namely, RNF8 has been shown to promote retention of NBS1 at LASER DNA damage (84), which as part of a complex with RAD50 and MRE11, can displace KU from DNA ends (85). Consistent with this notion, we found that NBS1 is important to suppress EJ without indels, and promote ALT-EJ (4-μHOM Embed assay), as mentioned above.

In the absence of KU70, RNF8 is not only dispensable for ALT-EJ, but inhibits this repair event, which indicates that RNF8 has an additional role in DSB repair that is independent of KU, which we identified as mediating HDR. Certainly, RNF8-mediated KU displacement could facilitate both ALT-EJ and HDR, but our findings indicate that RNF8 has a further role in HDR that is independent of KU. Consistent with this notion, RNF8 is important for recruitment of RNF168 to DNA damage (33,34), which has been shown recently to promote ubiquitination and recruitment of PALB2 to DNA damage (35–36,76). Furthermore, in cells that lack RNF168 and that are deficient for one allele of *Brca1*, RAD51 foci were shown to be rescued by a fusion protein between the RNF8 FHA domain to PALB2 (36). We found that a similar fusion protein could partially suppress the HDR defect in *Rnf8-/-* and *Rnf8-/-Ku70-/-* mESCs. These findings indicate that the KU-independent role of RNF8 in promoting HDR involves facilitating PALB2 function.

In performing the HDR experiments, we also noted differences between events induced by Cas9 versus I-SceI, which may have implications for gene editing mechanisms. Namely, loss of KU70 caused a greater increase in HDR events that were induced by I-SceI versus Cas9. One possible explanation for this result may be that Cas9 may be retained at DSB ends longer (86,87). Such Cas9 retention may delay KU binding, such that for some repair events, removal of Cas9 itself could be sufficient to initiate end resection for HDR. Thus, a subset of Cas9-induced HDR events may bypass the inhibitory effect of KU.

We also note that the reporter assay systems used in this study use either one or two Cas9 DSBs, which could influence the mechanism of repair. For all reporter assays measuring events requiring two DSBs (EJ6-GFP, EJ7-GFP, 4-μHOM, RMD-GFP), the rate of DSB repair could influence the probability that both DSBs occur simultaneously. Along these lines, the DSBs induced by nucleases are relatively persistent, because a likely outcome of DSB repair is precise EJ, which recreates the cleavage site that can be re-cut by the nuclease (88). Such persistent DSBs may be more prone to repair events that cause loss of the nuclease recognition site and/or increase the probability that two such DSBs occur simultaneously. Namely, use of nucleases to study DSB repair outcomes may cause a bias toward events that result in loss of the nuclease recognition site. However, we suggest that examining EJ between two DSBs without indels, with EJ7-GFP and/or amplicon sequencing of rearrangement junctions, provides a measure of a relatively precise repair event. Thus, while the persistent nature of nuclease-generated DSBs may bias outcomes toward mutagenic events, analysis of EJ between two DSBs without indels enables a comparison between repair events that protect the DSB ends from processing versus those that involve processing (i.e. ALT-EJ, mutagenic C-NHEJ, HDR and SSA).

In addition, for the reporters involving pairs of DSBs, some of the DSBs are in relatively close proximity (i.e. EJ7-GFP and 4-μHOM), and thereby may mimic clustered DSBs, which are a consequence of some forms of radiotherapy (89,90). Such clustered DSBs have been found to be prone to repair by ALT-EJ (91), which may be due to the reduced activity of KU on short DNA fragments to activate DNA-dependent protein kinase activity (89,90). Accordingly, EJ of multiple DSBs in close proximity may be prone to ALT-EJ repair (89,90). However, with the EJ7-GFP reporter, we observe a high frequency of EJ without indels (Figure 1C and Supplementary Figure S2), which indicates that C-NHEJ between two DSB is robust in this particular reporter design.

For the repair assays that involve multiple distant DSBs (i.e. the EJ deletion rearrangement measured by EJ6-GFP, and RMD-GFP), these events may require DSB mobility to enable the long-range interactions between two DSBs. Such DSB mobility is one proposed function of 53BP1 for mediating fusions of unprotected telomeres, and class switch recombination (92). Since RNF8 is important for 53BP1 recruitment to DSBs (20–22), one possible role of RNF8 could be to regulate such DSB mobility. For instance, since RNF8 inhibits a C-NHEJ-mediated deletion rearrangement (EJ6-GFP+TREX2), one possibility is that RNF8 suppresses DSB end mobility that is required for the long-range interactions of the DSB ends. However, RNF8 did not have an obvious effect on RMD events induced by two distant DSBs, and when the 3′ DSB was positioned relatively far from the repeat, which are events that also likely require long-range interactions. Thus, altogether, the findings support a role of RNF8 in promoting ALT-EJ/inhibiting C-NHEJ, rather than affecting DSB end mobility *perse*, as has been proposed as a function for 53BP1 (92).

Furthermore, we found key distinctions between RNF8 versus 53BP1, which we examined since RNF8 is critical for recruitment of 53BP1 to DNA damage, as mentioned above (20–22). Consistent with these factors functioning in the same pathway, both 53BP1 and RNF8 are important for two specialized EJ events: class switch recombination during lymphocyte development, and fusion of deprotected telomeres (44,46–47,53,93). However, both of these specialized EJ events can be mediated by either C-NHEJ or ALT-EJ (94,95), such that the precise role of 53BP1 in these EJ pathways has been unclear. Indeed, we find that 53BP1 does not have a major effect on EJ repair of Cas9 DSBs via C-NHEJ (EJ without indels) or ALT-EJ (4-μHOM Embed assay). These findings are consistent with a study of EJ repair of I-SceI DSBs, in that 53BP1 was shown to have no clear effect on EJ between two DSBs that are relatively close, and for repair between distant DSBs, 53BP1 is mostly important to suppress insertion mutations (96). Accordingly, 53BP1 is not specifically important for C-NHEJ or ALT-EJ, *perse*, such that the classification of 53BP1 as an NHEJ factor may be problematic. Rather, 53BP1 appears important for the specialized EJ events described above, which require synapsis of DSB ends across long-distances, as well as to regulate DSB end resection to favor HDR versus SSA (44–48). Furthermore, from our analysis of a *53bp1-/-Rnf8-/-* double mutant cell line, the influence of RNF8 on ALT-EJ appears independent of 53BP1.

Finally, we compared the influence of RNF8 versus POLQ during ALT-EJ, finding that POLQ remains important for ALT-EJ in KU-deficient cells. Indeed, consistent with recent reports (62,97–98), we found that combined disruption of POLQ and KU70 caused the loss of a diverse set of EJ events (i.e. EJ without indels, the 4-μHOM Embed EJ event and the 4-μHOM Terminal EJ event). Although, for EJ without indels, only expression of KU70, but not POLQ, was able to rescue this repair event. Conversely, expression of POLQ was more proficient for promoting the 4-μHOM Embed EJ event, compared to KU70. However, in the *Polq-/-Ku70-/-* mESCs, KU70 expression nonetheless caused a significant, albeit modest, increase in the 4-μHOM Embed EJ event. This finding supports the notion that KU70, and hence the C-NHEJ pathway, is capable of mediating EJ events involving end resection and annealing of microhomology (99), albeit at a much lower frequency that EJ without indels. Altogether, we have found that POLQ promotes ALT-EJ independently of KU, whereas the role of RNF8 in promoting ALT-EJ is dependent on KU, indicating that these factors have distinct/non-epistatic roles in genome maintenance, which is further supported by our inability to generate a *Polq-/-Rnf8-/-* double-mutant cell line. In conclusion, we suggest that RNF8 has KU-dependent (ALT-EJ) and KU-independent (HDR) roles in DSB repair.

## Supplementary Material

gkaa380_Supplemental_File
