# Supplementary material for: RNF8 has both KU-dependent and independent roles in chromosomal break repair
Source: Nucleic Acids Res. 2020 May 19;48(11):6032–52. doi: 10.1093/nar/gkaa380 (PMC7293022; doi:10.1093/nar/gkaa380)
Supplement: gkaa380_Supplemental_File [file gkaa380_supplemental_file.pdf]

SUPPLEMENTARY INFORMATION

RNF8 has both KU-dependent and independent roles in chromosomal break repair

L. Jillianne Tsai, Felicia Wednesday Lopezcolorado, Ragini Bhargava, Carlos Mendez-Dorantes, Eva Jahanshir, Jeremy M. Stark

Supplemental Table S1. Effects of siRNAs targeting 148 factors on the EJ6-GFP/TREX2 assay. Shown are the EJ6-GFP/TREX2 GFP+ frequencies normalized to siCTRL for the findings shown in Figure 1d, along with the siRNA catalog number from Dharmacon.

siRNA Catalog #	Gene Name	EJ6-GFP+TREX2 %GFP+ Normalized to siCTRL (mean)	N
M-040899-01	Nbn	2.950	10
M-040545-01	Brca1	1.910	10
M-048099-00	Rnf8	1.873	10
M-053978-01	Phf13	1.837	4
M-056219-01	Smarcad1	1.782	4
M-043653-01	Apaf1	1.733	4
M-040642-02	Trp53	1.727	2
M-047959-01	Rad50	1.659	4
M-042134-00	Top2b	1.602	4
M-058494-01	Wrn	1.538	4
M-059358-01	Rsf1	1.533	4
M-052542-01	Kdm4d	1.515	4
M-051344-00	Mre11a	1.437	4
M-047361-00	Cops3	1.435	2
M-059054-01	Ncl	1.419	4
M-044781-01	Pbrm1	1.412	2
M-058636-01	Cdkn1a	1.4	2
M-048839-01	Pnkp	1.4	2
M-058923-01	Vdr	1.4	2
M-045977-01	Strap	1.385	2
M-065688-01	Mdc1	1.371	2
M-061976-01	Bax	1.364	2
M-047837-01	Ccar2	1.363	4
M-040709-01	Akt1	1.3	2
M-044082-00	Msh6	1.3	2
M-040886-01	Srsf1	1.279	4
M-040827-01	Msh2	1.267	2
M-058043-01	Scai	1.257	4
M-047983-00	Rad18	1.235	2
M-047764-01	Nfkb1	1.226	4
M-058868-01	Nfat5	1.225	4
M-041733-00	Rnf20	1.2	2
M-049440-00	Sirt1	1.2	2
M-062394-00	Terf1	1.2	2
M-062621-00	H2afx	1.199	4
M-040028-01	Rif1	1.188	2
M-062727-01	Psme3	1.176	2
M-061277-01	Nfkbib	1.164	4
M-062864-01	Dna2	1.15	4
M-060591-01	Exo1	1.133	2

M-049293-01	Hmga1	1.133	2
M-057068-01	Cep164	1.125	4
M-046785-01	Dbf4	1.118	4
M-060281-01	Cbx1	1.114	2
M-042050-01	Atf1	1.111	2
M-053513-01	Dclre1c	1.111	2
M-042978-01	Bak1	1.091	2
M-058612-01	Bid	1.091	2
M-048005-00	Nabp2	1.083	2
M-049215-01	Lig3	1.077	2
M-048348-01	Terf2	1.077	2
M-043916-01	Top2a	1.077	4
M-046270-00	Xrcc1	1.077	2
M-053409-01	Fanci	1.067	2
M-049323-00	Mlh1	1.067	2
M-051629-00	Rrmb2	1.067	2
M-064311-01	H6pd	1.063	2
M-045651-01	Hspb1	1.063	2
M-041135-01	Smarca4	1.061	4
M-040544-01	Cdk5	1.056	2
M-057026-00	Paxip1	1.056	2
M-049351-01	Tdp1	1.056	2
M-041553-00	Fbxw7	1.043	2
M-058681-01	Eif43bp	1.027	4
M-059659-01	Aplf	1	2
M-045954-01	Aven	1	2
M-057795-01	Kat5	1	2
M-049373-01	Lig1	1	2
M-048269-01	Pmaip1	1	2
M-040150-00	Ripk1	1	2
M-057081-01	Slx4	1	2
M-055762-00	Smad1	1	2
M-042735-01	Traf6	1	2
M-065548-00	Usp28	1	2
M-065588-00	Mtch2	0.988	4
M-053878-00	Tinf2	0.962	4
M-056274-01	Mad2l2	0.957	2
M-044891-01	Siah1a	0.955	2
M-040135-00	Mapkapk2	0.938	2
M-050032-00	Bbc3	0.933	2
M-056921-01	Fancd2	0.933	2
M-043585-01	Hmga2	0.933	2
M-049483-00	Smc1a	0.929	10
M-048587-00	Nono	0.923	2
M-050773-00	PolQ	0.917	4
M-051908-01	Wrap53	0.913	2
M-061987-01	Blm	0.909	2
M-066282-00	Chd3	0.891	4
M-040800-01	Trim28	0.879	4

M-050929-01	Upf1	0.878	4
M-056772-01	Lig4	0.877	4
M-171120-00	LOC	0.875	2
M-040959-01	Creb1	0.867	2
M-041301-01	Herc2	0.867	2
M-040114-01	Hipk2	0.867	2
M-063467-01	Lats1	0.864	2
M-043871-01	Trp73	0.864	2
M-062123-01	Mcm2	0.85	4
M-040796-01	Ikbkg	0.846	2
M-055649-01	Dck	0.834	4
M-063913-01	Fbxo31	0.833	2
M-042671-01	Mcm3	0.833	2
M-060606-01	Chaf1a	0.818	2
M-058766-01	Mdm4	0.818	2
M-063242-01	Rassf1	0.818	2
M-052244-01	Usp7	0.818	2
M-062189-01	Hus1	0.813	2
M-043543-01	Cdc6	0.8	2
M-058640-02	Chek1	0.8	2
M-050322-01	Dclre1c	0.8	2
M-040023-00	Parp1	0.778	2
M-040657-00	Ppp2ca	0.778	4
M-063609-01	Uimc1	0.769	2
M-046515-01	PolI	0.765	2
M-044993-03	E2f1	0.75	2
M-040125-01	Mapk14	0.75	2
M-048531-01	Pcna	0.75	2
M-042850-00	Polb	0.75	2
M-043783-01	Rad17	0.75	2
M-040058-01	Ppp4c	0.744	4
M-041938-00	Mcph1	0.743	2
M-040604-01	Chek2	0.733	2
M-058585-01	Ppp2r5c	0.733	2
M-043438-02	Rfwd2	0.733	2
M-064492-01	Smc3	0.733	10
M-049191-01	Cdc25c	0.721	4
M-051582-01	Csnk2a2	0.714	2
M-048649-01	Pias4	0.692	2
M-042321-00	Clspn	0.686	4
M-050145-01	Pdp1	0.667	2
M-042308-01	Rad9a	0.667	2
M-044342-01	Stk11	0.642	4
M-042505-00	Gadd45	0.639	4
M-040440-01	Stk3	0.613	4
M-041016-01	Xrcc4	0.611	4
M-040054-01	Ppm1g	0.6	2
M-040633-02	Sp1	0.568	4
M-062739-01	Rpa2	0.567	10

M-040815-01	Setdb1	0.533	2
M-065607-01	Ep300	0.472	4
M-041098-01	Mdm2	0.428	4
M-063535-01	Eif3e	0.391	2
M-046247-01	Yap1	0.387	4
M-045791-01	Rps6	0.231	2
M-050241-01	Gmnn	0.167	2
M-048366-01	Cdca5	0.1	2
M-040566-01	Plk1	0.086	2
M-057592-01	Vcp	0	2

Supplemental Table S2. Generating a *Polq-/-Rnf8-/-* mESC line appears unfeasible. WT and *Polq-/-* mESCs were transfected with expression vectors for Cas9/sgRNAs targeting two DSBs in *Rnf8*, and individual clones were screened for a deletion by PCR (see Supplemental Figure S1). Shown are the number of clones with homozygous *Rnf8* WT, heterozygous *Rnf8* WT/deletion, and homozygous *Rnf8* deletion. Number of clones with the different genotypes for WT vs. *Polq-/-* statistically different based on chi-squared analysis, P<0.0001.

	WT *Rnf8*	Heterozygous *Rnf8* (WT/deletion)	Homozygous *Rnf8* deletion	% Homozygous *Rnf8* deletion
WT	29	8	10	21.3
Polq-/-	197	2	0	0

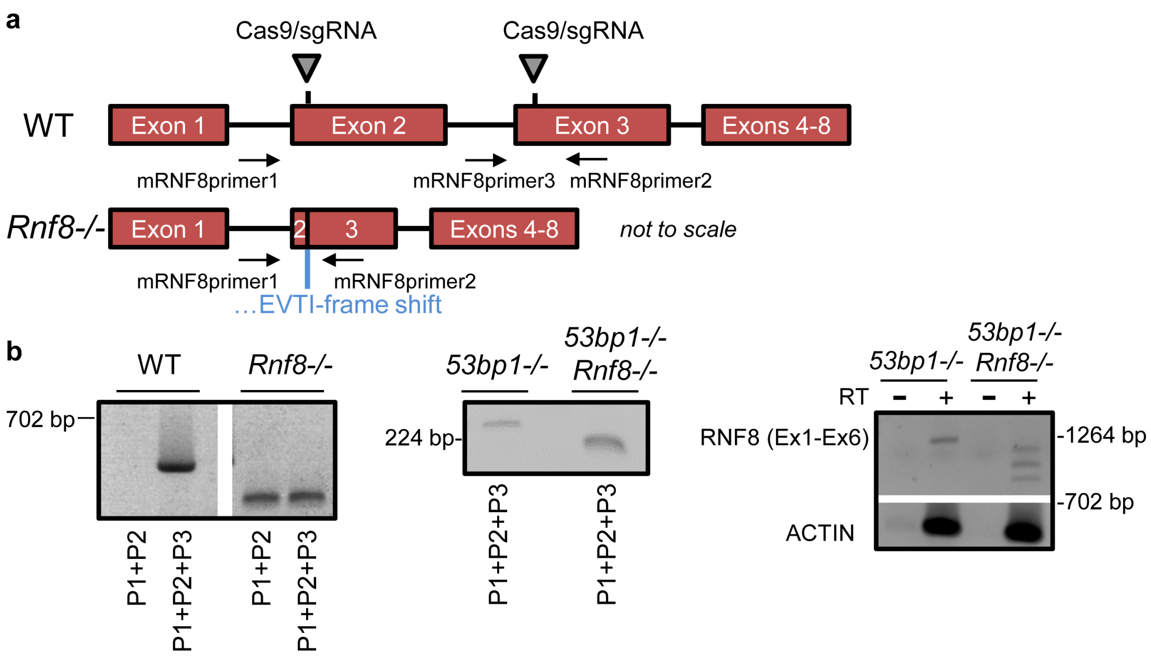

Supplemental Figure S1. Generation of *Rnf8-/-* mESC lines. (a) Shown is a schematic of mouse *Rnf8* exons (not to scale). Two sgRNAs were used to target *Rnf8*: one in Exon 2, and one in Exon 3. Sequencing analysis revealed that the *Rnf8-/-* mESC line contains the frame shift shown. **(b)** Genotyping of the *Rnf8* alleles using two primer sets, shown in (a) (not to scale). The first set uses mRNF8primer1 and mRNF8primer2 (P1+P2). The second set uses mRNF8primer1, mRNF8primer2, and mRNF8primer3 (P1+P2+P3). Primers P2+P3 amplify only WT *Rnf8* and this PCR product is larger than that of the deletion mutation amplified by primers P1+P2. Also shown is an RT-PCR amplification product for a region of the *Rnf8* mRNA from exons 1-6 (Ex1-6), and Actin control, for RNA from *53bp1-/-* and *53bp1-/-Rnf8-/-* mESCs, treated with and without reverse transcriptase (RT).

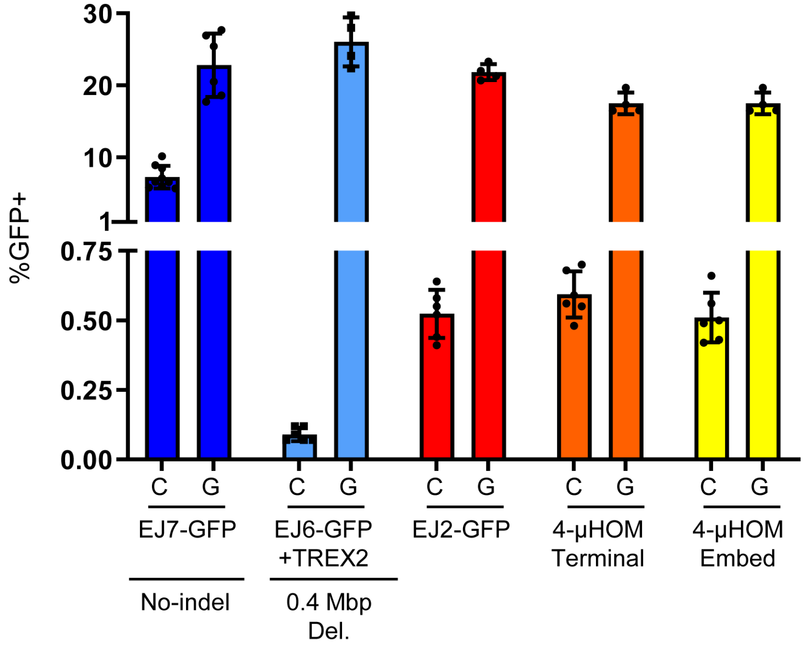

Supplemental Figure S2. GFP+ frequencies of multiple reporter assays without normalization to transfection efficiency, which is shown separately. Shown are the GFP+ frequencies for several chromosomal reporter assays (EJ7-GFP, EJ6-GFP+TREX2, EJ2-GFP, 4-μHOM Terminal, and 4-μHOM Embed) in WT mESCs treated with siCTRL, for the data shown in Figure 2b. Frequencies are for transfections of sgRNAs/Cas9 (Cas9, C) or the GFP expression vector pCAGGS-NZE-GFP (GFP, G). Error bars represent SD. N=6 for sgRNAs/Cas9 and N=4 for pCAGGS-NZE-GFP, except in EJ7-GFP which is N=9 for sgRNAs/Cas9 and N=6 for pCAGGS-NZE-GFP.

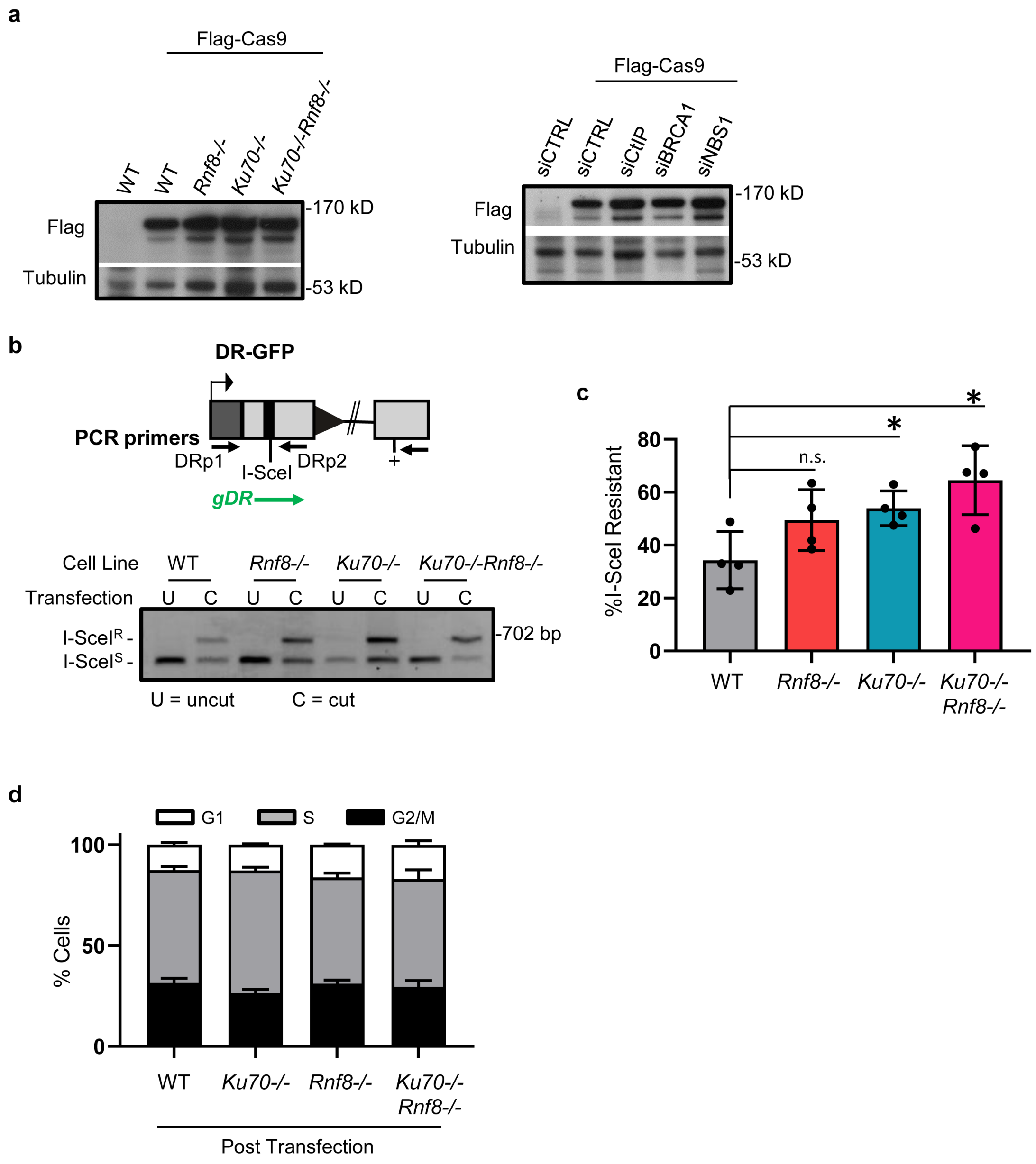

Supplemental Figure S3. Cas9 and cell cycle control experiments. (a) Flag-Cas9 expression. The Cas9/sgRNA expression vector used in this study uses Cas9 with a Flag immunotag. Shown are immunoblots for Flag and Tubulin control for WT, *Rnf8-/-*, *Ku70-/-*, and *Ku70-/-Rnf8-/-* mESCs (left), and WT mESCs treated with siCTRL, siCtIP, siBRCA1, or siNBS1 (right), each transfected with Cas9/sgRNA plasmids as for the reporter assays, except including a puromycin resistance expression plasmid, and puromycin selection to enrich for transfected cells. Untransfected WT is shown as a control. **(b)** I-SceI site loss assay. Shown is a diagram of DR-GFP with PCR primers (DRp1 and DRp2) flanking the I-SceI recognition site, along with the sgRNA targeting the I-SceI site (gDR) (not to scale). Also shown is a representative image of I-SceI^{R} (resistant) and I-SceI^{S} (sensitive) PCR products in WT, *Rnf8-/-*, *Ku70-/-*, and *Ku70-/-Rnf8-/-* mESCs either untransfected (U), or transfected with the sgRNAs/Cas9 (Cas9, C) expression

vector targeting DR-GFP, and which were digested with I-SceI. **(c)** I-SceI site loss, following transfection of the sgRNA/Cas9 expression vector, is not statistically different between WT and *Rnf8-/-* mESCs, but is elevated in KU70-deficient cells. Shown is the percentage of I-SceI^R PCR products in WT, *Rnf8-/-*, *Ku70-/-*, and *Ku70-/-Rnf8-/-* mESCs, transfected and analyzed as in (b). Error bars represent SD. N=4. (*) P≤0.042 using unpaired multiple t-tests with the Holm-Sidak correction. **(d)** Loss of KU70 and/or RNF8 does not have obvious effects on cell cycle phase post-transfection. Shown is the frequency of cells in G1, S, and G2/M, for WT, *Ku70-/-*, *Rnf8-/-*, and *Ku70-/-Rnf8-/-* mESC post-transfection with an expression plasmid for sgRNA/Cas9 and a puromycin resistance plasmid to enrich for transfected cells. Puromycin selection was added the day after transfection, and BrdU labeling and fixation was two days after transfection. N=3. Error bars represent SD.
